# SuperTAD: robust detection of hierarchical topologically associated domains with optimized structural information

**DOI:** 10.1186/s13059-020-02234-6

**Published:** 2021-01-25

**Authors:** Yu Wei Zhang, Meng Bo Wang, Shuai Cheng Li

**Affiliations:** Department of Computer Science, City University of Hong Kong, 83 Tat Chee Ave, Kowloon Tong, Hong Kong, China

**Keywords:** Topologically associating domain, Hi-C, Structure information theory, Dynamic programming

## Abstract

Topologically associating domains (TADs) are the organizational units of chromosome structures. TADs can contain TADs, thus forming a hierarchy. TAD hierarchies can be inferred from Hi-C data through coding trees. However, the current method for computing coding trees is not optimal. In this paper, we propose optimal algorithms for this computation. In comparison with seven state-of-art methods using two public datasets, from GM12878 and IMR90 cells, SuperTAD shows a significant enrichment of structural proteins around detected boundaries and histone modifications within TADs and displays a high consistency between various resolutions of identical Hi-C matrices.

**Supplementary Information:**

The online version contains supplementary material available at (10.1186/s13059-020-02234-6).

## Background

The 3D architecture of chromatin plays vital roles in DNA replication and gene transcription process. Many techniques have been devised to capture the architectural information within whole genome [1–9], among which the High-throughput Chromosome Conformation Capture (Hi-C) technique has gained widespread adoption. Hi-C applies high-throughput sequencing to collect fragments that are ligated due to spatial proximity within the genome. Well-established procedures can aggregate and transform sequenced reads into a Hi-C matrix (called *contact map*) at a specific resolution. An element in the Hi-C matrix represents the contact frequency between two fixed-size genome regions (referred to as *bins* or *windows*) of which the indices correspond to the row and column indices in the matrix.

Contact maps have enabled the discovery of architectural units within a chromatin, called *topologically associating domains* (TADs) [10–12]. Genomic regions within a TAD interact with each other more intensely than those between different TADs. TAD can be further subdivided into sub-topologies (sub-TADs), and proximal TADs can aggregate into a higher-order structural domain (meta-TAD), resulting in a hierarchy of the TADs. The size of a TAD varies from thousands of base pairs (kbps) to several million base pairs [10]. TADs are the basic unit of both nucleus conformation and gene regulation [11, 13–16]. The boundary between TADs can obstruct the spread of activity and has been shown to enrich inhibiting factors such as CTCF binding sites, cohesin complexes, and housekeeping gene TSSs, SINE retrotransposons [10, 13, 16–18]. More significantly, the alteration of certain TAD boundaries can lead to cancers or developmental disorders [19–23].

Determining TAD boundaries from Hi-C data remains a challenging task [24]. Some computational methods for detecting the hierarchical structure of TADs exist [25–27]. In 2014, Rao et al. [28] proposed Arrowhead algorithm to transform the square domain feature into an arrowhead-shaped feature and detect TAD through corner score. Each TAD is determined independently, and some of them show a hierarchy manner. TADtree [29], the first published algorithm to identify hierarchy, assumes that the signals of the background and TADs are linear. It models and captures the hierarchical structure of TADs with a forest. Haddad et al. [30] proposed a hierarchical clustering approach named IC-Finder for reconstructing TADs. Yu et al. [31] introduced a Gaussian Mixture model And Proportion test (GMAP) algorithm, which is iteratively applied to normalize the Hi-C matrix until no elements of the statistical test are significant, or the domain size is smaller than a pre-specified threshold. Norton et al. [32] proposed a graph-theory-based method named 3DNetMod to detect TADs by maximizing network modularity. Li et al. [33] proposed a method named deDoc that interprets Hi-C matrix as a weighted graph. Then, the problem is to find a partition with minimal structural information (entropy). They proposed a method which, through a top-down greedy recursion of partitioning and clustering, produces a hierarchical structure (called a *coding tree*) of TADs with the minimal *structural entropy*. The algorithm is heuristic and does not guarantee optimal results. As both the graph partitioning and clustering problems are NP-complete, it is hard to obtain a coding tree of minimal structural entropy. Nevertheless, the TAD boundaries inferred by deDoc demonstrated high consistency with Hi-C matrices at different resolutions. This shows structural information theory to hold promises in the discovery of the TADs. The recent proposed OnTAD algorithm [34] applied dynamic programming to identify the TADs from candidate boundaries, which recursively partitioned the genome while maximizing a score function that depicts the contact frequency inside the TAD hierarchy.

In this work, we design optimal algorithms for computing the coding tree of a contact map. While the problem of finding an optimal tree from a general graph is NP-hard, we observe that the graphs which correspond to the contact maps possess specific properties that allow efficient algorithms for finding their coding trees. One such property is that the vertices in a contact map are ordered. As a result, the leaf nodes of the coding tree form a partition of the bins according to the order. Here, we prove that the problem is polynomial-time solvable. Also, we prove a unique property that can significantly reduce the search space. We designed an optimal algorithm using dynamic programming with polynomial time for computing the coding tree of a Hi-C contact map with minimal structural information. We implemented the algorithms into a software package named SuperTAD.

We compare our method with seven existing methods that can infer the TAD hierarchy, namely Arrowhead, TADtree, IC-Finder, GMAP, 3DNetMod, deDoc, and OnTAD (Table 1). The results reveal that the TADs detected by SuperTAD have minimal average structure entropy and the highest average contact density, as well as the highest enrichment of structural proteins at boundaries and histone modifications within TADs. The results of SuperTAD under various resolution matrices give the highest agreement (the average overlapping ratio is 0.945 for GM12878 cell line, and 0.95 for IMR90 cell line across 25 kb vs. 50 kb and 50 kb vs. 100 kb).

**Table 1:** Properties of methods for detecting hierarchies of TADs

	SuperTAD	OnTAD	deDoc	3DNetMod	GMAP	IC-Finder	TADtree	Arrowhead
Last updated	2020	2019	2018	2018	2017	2017	2016	2014
Journal	–	*Genome Biology*	*Nature Communications*	*Nature*	*Nature Communications*	*Nucleic Acids Research*	*Bioinformatics*	*Cell*
Input	Raw/normalized	Raw/normalized	Raw/normalized	Normalized	Normalized	Raw/normalized	Normalized	Raw/normalized
Model	Structural information theory,	Adaptive local minimum search,	Structure information theory,	Network modularity maximization,	Gaussian mixture model,	Hierarchical clustering,	Boundary index (BI),	Arrowhead transformation
	dynamic programming	contact density signal maximization	greedy merging and combining	hierarchical spatial variance minimization	proportion test	local directionality index (DI)	dynamic programming,	
							weighted interval scheduling with	
							multiplicities (WISM)	
Levels of TAD	Not limited	Not limited	2	Not limited	Controlled by parameter	Controlled by parameter	Not limited	Not limited
# Parameters	0	2	0	5	4	1	6	1
Reference	–	[34]	[33]	[32]	[31]	[30]	[29]	[28]

## Results

### Overview of SuperTAD

**Fig. 1:**
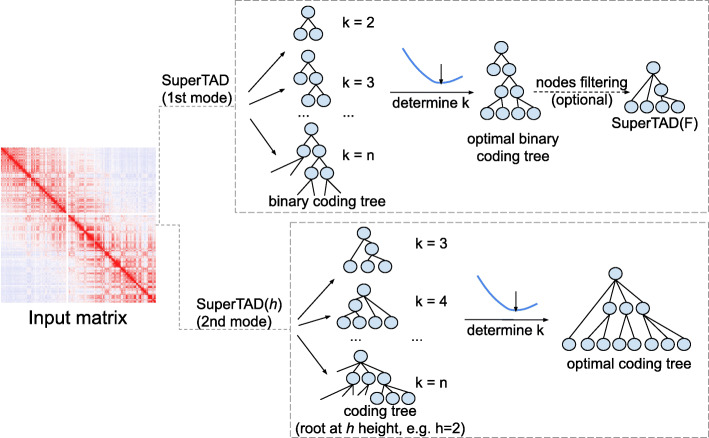
The overview of SuperTAD pipeline. With the same input matrix, SuperTAD provides two modes for users. SuperTAD (the first mode) does not require any user-defined parameter and can determine the height of the coding tree by self-learning. SuperTAD(*h*) (the second mode) receives the manually selected *h* as the only parameter and finds the optimal coding tree with the constraint of *h*. For both modes, many coding tree candidates with various leaves number *k* are created. The optimal coding tree is selected by determining the most appropriate *k*. For SuperTAD, optional node filtering is performed to prune false-positive TADs from the optimal binary coding tree. The result after pruning is referred to as SuperTAD(F)

SuperTAD implements two variants of our algorithms, one which requires a pre-specified threshold, and one without such a requirement. Both variants find optimal coding trees from a contact map. SuperTAD is an open-source, written in C++, and runs from the command line. It accepts either raw or normalized Hi-C matrix as input (Fig. 1). Given an input matrix, SuperTAD provides two modes for users, corresponding to the two implemented variants. If a user supplies an integer parameter *h*, it will construct the optimal coding tree of height at most *h*, as SuperTAD(*h*). Otherwise, it will construct the optimal tree among all the possible heights. Given an optimal coding tree, we provide a filter to the tree nodes, pruning away non-TAD ones, resulting only in TAD nodes.

To evaluate the similarity between two coding trees *T* and *T*^′^ resulted from the same contact map, we propose a symmetry metric called *overlapping ratio*, which measures the maximum intersection between two results. In our work, we use the overlapping ratio, an asymmetry metric weighted similarity proposed by Li et al. [33] as well as the measure of concordance (MoC) proposed by Zufferey et al. [27] to quantify the level of agreement in the results called through different methods.

### Comparison with deDoc using simulation data with various noise ratios and sizes

As deDoc and SuperTAD both apply structural information theory, it is interesting to know if they lead to divergent results. We first compared their relative accuracy and robustness. To better quantify the performance, we used simulated data with various noise ratios and TAD sizes and independently executed both approaches 100 times under each setting. As input, we generate an adjacent matrix *A*={*a*_*ij*_}_*N*×*N*_,∀*a*_*ij*_∈{0,1} representation for each graph *G*={*V*,*E*}. *G* contains non-overlapped clusters, and the number of clusters is fixed. If an edge (*v*_*i*_,*v*_*j*_)∈*E*(*G*), then *a*_*ij*_=1; otherwise, *a*_*ij*_=0.

To quantify the accuracy and the quality of the results, we compared the overlapping ratio, the weighted similarities between the real structure (reflected in simulated data), and the inferred structures from deDoc or SuperTAD. As weighted similarity is asymmetry, we set *X* in $ws_{X}^{Y}$ to the actual structure, and let *Y* be the result of deDoc or SuperTAD. We calculated the structural entropy of the coding tree for every result.

First, we examined the influence of noise in the performance of both deDoc and SuperTAD. We fixed the probability of intra-interaction in each cluster and set the size of all clusters (the number of vertices contained in the cluster) to be equal. Then, we tested the probability of inter-interaction from 5 to 50% by 5% of the probability of intra-interaction (denoted as noise ratio). A higher noise ratio implies more edges across different clusters. For each noise ratio, we simulated a matrix as input to both algorithms. This is repeated 100 times. The simulated structure is a set of non-overlapped TADs (i.e., all at one level). Hence, we only assessed the result of deDoc(E), which is the case where there is only one layer of clusters.

Statistical results of repeated experiments show that the interactions across the clusters have a greater influence on deDoc than SuperTAD. Before the noise ratio reaches 40%, the overlapping ratio and weighted similarity between actual structure and results of SuperTAD are close to 1 (Additional file 2: Figure S6a, blue boxes), suggesting a high level of noise tolerance in SuperTAD. For deDoc, the overlapping ratio and weighted similarity between true structure and result of deDoc began to decrease when noise ratio reaches 15% (Additional file 2: Figure S6a, orange boxes). Note that the median value (Additional file 2: Figure S6a, black solid line in the boxes) of deDoc boxes reaches 0 when the noise ratio is equal to 40%. That is, deDoc failed to discover any cluster from the input matrix. The structural entropy of coding tree detected by both algorithms demonstrated similar distributions (except for some outliers of deDoc) at 5% noise ratio. This shows that both algorithms can detect the clusters with high accuracy when there are few inter-domain interactions. However, at a higher noise ratio, deDoc’s solutions have higher structural entropy than SuperTAD’s in general.

Next, we examined how the methods perform under different standard deviations on the TAD sizes. We fixed the probability of intra-interaction in each cluster, with a noise ratio of 10%, and assumed the sizes to obey a Gaussian distribution with the same mean. We performed 100 tests with each standard deviation in {1, 2, 3, 4, 5}. For both algorithms, the solutions started to deviate from the true structure when the standard deviation is at or above 3, with deDoc deteriorating faster than SuperTAD (Additional file 2: Figure S6b). As with the previous tests, deDoc’s solutions demonstrated higher structural entropy than SuperTAD’s.

### Comparing SuperTAD(2) with deDoc using real Hi-C matrix

In addition to the tests using simulated data, we tested the algorithms with real Hi-C matrices. As deDoc detects TADs with only two levels, we tested it against the second mode of SuperTAD (SuperTAD(*h*)), setting *h* to 2 (referred to as SuperTAD(2)). We downloaded the two in situ Hi-C processed contact datasets (.hic format) from Rao et al. [28]. Both datasets are combined across replicates and filtered with MAPQ ≥ 30. In the comparison between SuperTAD(2) and deDoc, we selected two bin resolutions 25 kb and 50 kb for assessing the robustness of the algorithms at various resolutions. The raw matrices were normalized with Juicer built-in Knight-Ruiz normalization into normalized Hi-C matrices (referred to as *KR matrix*).

First, we evaluated SuperTAD(2) and deDoc with identical matrix at bin resolution of 25 kb. We compared the distribution of length (size), structural entropy, and contact density of TADs inferred through both methods (Additional file 3: Figure S7a–c). The contact density is defined as the count of intra-TAD contacts divided by TAD length [33]. Compared to deDoc, TADs of SuperTAD(2) have a higher mean and median value in length, structural entropy, and contact density (the solid line in the box corresponds to the median while the dashed line and number in red corresponds to the mean). Next, we compare the structure entropy of the coding tree across various cell lines and bin resolutions. As shown in Additional file 1: Figure S3d, SuperTAD(2)’s solution always has less structure entropy for each comparison, which indicates SuperTAD(2) encoded the input data with lower uncertainty.

It has been previously reported that TAD boundaries are positively associated with the enrichment of the CCCTC-binding factor (CTCF) and members of the cohesin protein complex, such as RAD21 and SMC3 [10, 13]. We downloaded the IDR peaks data of transcription factor (TF) ChIP-seq from ENCODE and computed the fold change of peak enrichment between TAD boundaries and background for each structural protein (see the “Methods” section). We noticed that the boundary inferred by SuperTAD(2) has a greater fold change than deDoc for both cell lines (Additional file 3: Figure S7e)

Considering the fact that some Histone H3 modifications indicating the transcriptional activity are associated with TADs, we next evaluated the enrichment of Histone H3 modifications within detected TADs. We chose the repressing (H3K27me3) and activating (H3K36me3) marks as they exhibit a good mutual exclusion and can indicate either active or repressed transcriptional domains on a well mappable part of the genome [11, 12, 28, 35]. We downloaded the fold change over control data of ChIP-seq from ENCODE and calculated the observed average log10 ratio (LR) of H3K27me3/H3K36me3 and the empirical *p* value for each TAD. Based on the FDR-corrected *p* value, we identified the TADs that significantly enriched for either mark (FDR-corrected *p* value ≤0.1) from those with no significant enrichment (FDR-corrected *p* value >0.1). Then, based on the observed LR value, we further divided the TADs from the former group (FDR-corrected *p* value ≤0.1) into two sets, one is enriched in H3K27me3, the other is enriched in H3K36me3. The TADs inferred by SuperTAD(2) have a higher fraction of TADs enriched for histone modifications than deDoc (Additional file 3: Figure S7f).

We evaluated the robustness of the algorithms by comparing the similarity of the detected TADs for the same cell type across different bin resolutions. Resultant heatmaps for the 25-kb and 50-kb resolution matrix for both cell lines are shown with the detected TAD boundaries in Additional file 3: Figure S7g and h. To better quantify the similarity, we calculated the overlapping ratio, weighted similarity, and the MoC between the two results (Table 2). The result shows that the agreement of SuperTAD(2) is lower (− 0.02) for GM12878 cells and higher (+ 0.08) for IMR90 cells than deDoc across all metrics.

**Table 2:** Assessment of similarity criteria between the results of various resolutions and raw/normalized matrix

			SuperTAD(2)	deDoc
GM12878	25 vs. 50 (kb)	OR	0.81	red0.83
		WS	0.85	red0.87
		MoC	0.71	red0.73
	Raw vs. KR (average between 25 and 50 kb)	OR	red0.92	0.83
		WS	red0.93	0.88
		MoC	red0.90	0.77
IMR90	25 vs. 50 (kb)	OR	red0.88	0.79
		WS	red0.90	0.83
		MoC	red0.75	0.67
	Raw vs. KR (average between 25 and 50 kb)	OR	red0.93	0.88
		WS	red0.95	0.91
		MoC	red0.89	0.83

The larger value is labeled in red for each line

*OR* overlapping ratio, *WS* weighted similarity, *MoC* measure of concordance

As deDoc has been reported to work well with raw Hi-C matrices (i.e., without normalization), we performed further experiments to assess the similarity between results from raw and KR matrices. The heatmaps with the detected boundaries from both raw and KR matrices are shown in Additional file 3: Figure S7g, h. The overlapping ratio, weighted similarity, and MoC are as shown in Table 2. The comparison shows that compared to deDoc, SuperTAD(2) has higher consistency between its results from raw and KR matrices.

### Comparison of SuperTAD with existing methods for detecting hierarchies of TADs

The results thus far suggest that, under the two-layer constraint, SuperTAD(2) performs better than deDoc in terms of accuracy and robustness. We further investigate the performance of SuperTAD without the constraint. Many methods are able to determine the number of layers to use naturally from the input matrix. SuperTAD is able to do the same in the first mode. We compare SuperTAD (the first mode) with seven existing methods, namely OnTAD, deDoc, 3DNetMod, GMAP, IC-Finder, TADtree, and Arrowhead (Tabel 1). The analysis is performed on Hi-C data sets of two human cell lines (GM12878 and IMR90), the same as the last section. We selected a segment from chromosome 6 (Chr 6: 20000.0–30000.0 kb) for evaluation and comparison among all the methods.

#### Comparison of length, structural entropy, and contact density of inferred TADs

**Fig. 2:**
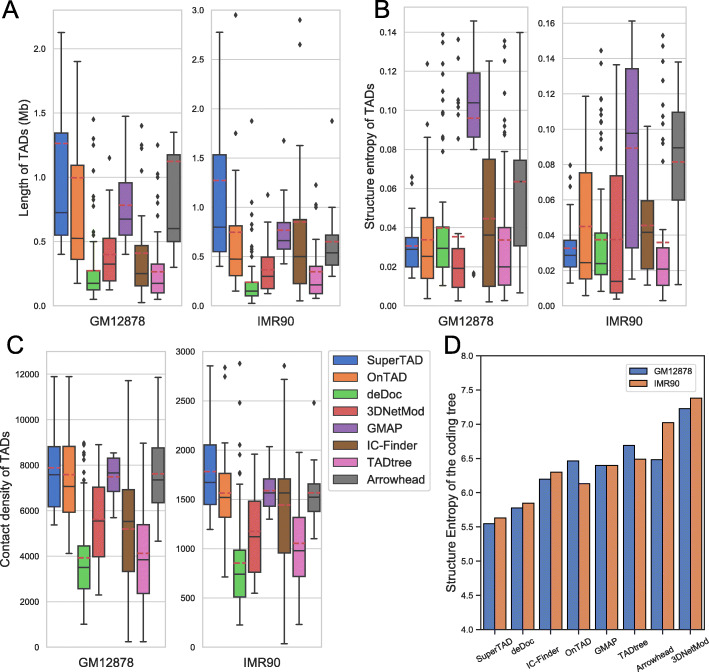
The statistics of TAD attributes for all the methods. We apply all the methods on KR (normalized) Hi-C matrix at 25-kb bin resolution for two human cell lines (GM12878 and IMR90). The boxplots show the statistics on **a** length, **b** structural entropy, and **c** contact density of TADs detected by all the methods. Each boxplot shows the value distribution of each method for each cell line, and all share the legend in **c**. The dashed lines in red and the solid lines indicate the mean and median value for each box. **d** The total structural entropy of the coding tree for each method. A lower bar corresponds to a coding tree with less structural entropy

We first compared the distribution of length (size), structural entropy, and density of the TADs from each method. As input, we use KR contact map at 25-kb bin resolution. Note that when constructing the coding tree for calculating structure entropy of the TADs for Arrowhead and 3DNetMod, we discarded the small TADs that are incompatible with the formed coding tree (these methods allows for overlapping across TADs, which conflict with the definition of the coding tree). Additionally, we use all the identified TADs from Arrowhead and 3DNetMod for the other analysis. The length of TADs inferred by SuperTAD has a broader range for both cell lines (Fig. 2a), which agrees with the hierarchical property of TADs. The TADs inferred by SuperTAD have the minimal mean value (the dashed line in red in boxes) of structural entropy for both cell lines (Fig. 2b). OnTAD and deDoc have a median value of structural entropy similar to SuperTAD but also a higher variance. SuperTAD has the highest mean value of contact density for both cell lines and the highest median value for IMR90 cells. OnTAD, GMAP, and Arrowhead also have a higher mean value of contact density for both cell lines (Fig. 2c). The conserved performance of SuperTAD between both cell lines proves that the TADs inferred by our method are highly self-dense structures.

To assess the uncertainty embedded in the detected coding tree, we further computed the structural entropy of the whole coding tree for each method. SuperTAD gives the coding trees with the minimum structural entropy for both cell lines (Fig. 2d), with deDoc ranked in the second place.

#### Significant enrichment of epigenetic characteristics at SuperTAD-detected boundaries or within TADs

Growing evidence shows that TAD may serve as the fundamental unit of gene regulation. The boundaries between TADs can obstruct the spread of activity within the genome. To validate the TAD boundaries inferred from each method, we downloaded the IDR peaks data of Transcription Factor (TF) ChIP-seq from ENCODE (https://www.encodeproject.org/) and computed the fold-change enrichment between detected boundaries and the background region. The data include the CCCTC-binding factor (CTCF), RAD21, and SMC3.

**Fig. 3:**
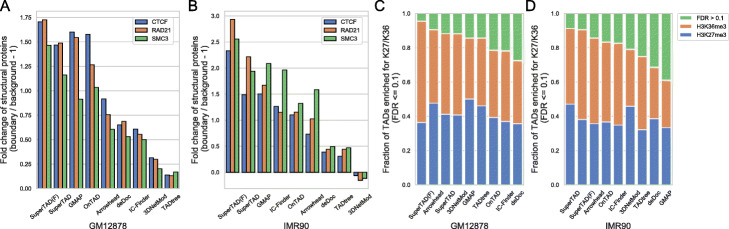
The enrichment of epigenetic characteristics at detected boundaries or within TADs for each method. **a**, **b** The fold change of structural proteins peak number (CTCF, RAD21, SMC3) between peaks (regions around boundaries) and background (regions located 400 kb away from the boundaries) for **a** GM12878 cells and **b** IMR90 cells. The methods in *x*-axis are ordered based on the average fold change across all the structural proteins for each cell line. **c**, **d** The cummulative bar diagram shows the fraction of TADs from three groups: enriched for H3K27me3 (FDR-corrected *p* value ≤0.1, the blue bar); enriched for H3K36me3 (FDR-corrected *p* value ≤0.1, the orange bar); no significant enrichment (FDR-corrected *p* value >0.1, the green bar). The methods in *x*-axis are sorted in the ascending order of the faction of the third group (no significant enrichment, FDR >0.1) for **c** GM12878 cells and **d** IMR90 cells. Note that we add both SuperTAD and SuperTAD(F) into the comparison, representing the results before and after the node filtering, respectively

We computed the average number of peaks for each structural protein for every 5 kb. The region around the identified TAD boundary (± 1 bin) are referred to as peaks while the 100-kb-length region located 400 kb away from the boundaries at both sides are referred to as background. We then calculated the ratio between the average number of peak and background and then let fold change as the ratio −1. We also added the selected TADs after pruning the optimal coding tree (referred to as SuperTAD(F)) into the comparison. The selected TADs have a higher structure entropy than their parent and a high probability of being self-dense from a random experiment with 1000 times simulations. As shown in Fig. 3a, SuperTAD(F) has the greatest fold change for all the structural proteins, while SuperTAD ranks the second. The results on the IMR90 dataset have the same trend (Fig. 3b).

To quantify the enrichment of Histone H3 methylation marks within TADs, we computed the observed average log10 ratio (LR) between H3K27me3/H3K36me3 as well as the empirical *p* value. Based on the FDR-corrected *p* value and observed LR value, we classified the TADs into three groups, TAD enriched for H3K27me3 (FDR-corrected *p* value ≤0.1), TAD enriched for H3K36me3 (FDR-corrected *p* value ≤0.1), and neither (FDR-corrected *p* value >0.1). SuperTAD(F) shows the minimal fraction of the TADs that enriched for neither of the histone marks for GM12878 cells (Fig. 3c). SuperTAD ranks the second for GM12878 cells, while the first for IMR90 cells. Note that both SuperTAD and SuperTAD(F) exhibit a consistent performance in the overall enrichment comparisons, indicating the TAD inference’s validity.

#### High consistency between SuperTAD results at different resolutions

To assess the consistency of the results’ output by the algorithms at various resolutions of identical data, we tested them using Hi-C matrices at 25-kb, 50-kb, and 100-kb bin resolutions.

We show the Hi-C matrices’ heatmap with the inferred boundaries of both results at 25 kb vs. 50 kb in Additional file 4: Figure S8 (for 50-kb vs. 100-kb heatmap, see Additional file 1: Figure S1). As can be observed, SuperTAD, SuperTAD(F), and deDoc are relatively consistent on both the GM12878 (the top line) and the IMR90 (the bottom line) cell lines. Arrowhead has a higher divergency for IMR90 than GM12878 cells, indicating it suffers from the relative lower depth of the data. TADtree has many duplications around the boundaries, resulting in vast disagreement between its results at different resolutions. OnTAD and IC-Finder show similar across 25 kb and 50 kb for GM12878 cells. However, their identified TADs at higher levels show high divergency in IMR90 cells, which implies that both OnTAD and IC-Finder are susceptible to the depth of data. For GMAP, the size of TADs inferred at 50-kb resolution is much larger than that at 25-kb resolution for both cell lines (GMAP fails to detect TADs from the identical input at 100-kb resolution). In this test, 3DNetMod performed the poorest. Its result at 25-kb resolution has as many duplications around the boundaries as TADtree, and it was unable to detect any boundary at 50-kb (as well as 100 kb) resolution (since 3DNetMod filters out all the regions when detecting “good regions,” and the algorithm cannot determine the value of maximum gamma afterward).

We show in Table 3 the overlapping ratio, weighted similarity, and MoC for the similarity quantitative assessment. We compute the agreement of 25 kb vs. 50 kb and 50 kb vs. 100 kb. Among all the methods, SuperTAD shows the highest consistency in its results at various resolutions. Node filtering in SuperTAD(F) degrades the consistency to some extent for both cell lines (SuperTAD(F) ranks the second for GM12878 cells and the third for IMR90 cells). deDoc and IC-Finder also show high agreement across resolutions through overlapping ratio and weighted similarity for both cell lines. As both SuperTAD and deDoc are structural information theory-based algorithms, self-consistency at different resolutions may be advantageous for this approach.

**Table 3:** Assessment of similarity criteria between results of 25-kb vs. 50-kb and 50-kb vs. 100-kb resolutions for all methods

			SuperTAD	SuperTAD(F)	OnTAD	deDoc	3DNetMod	GMAP	IC-Finder	TADtree	Arrowhead
GM12878	25/50^1^	OR	red0.96	red0.93	0.91	0.88	N/A^2^	0.68	0.92	0.64	0.64
		WS	red0.97	red0.96	0.94	0.90	N/A	0.78	0.94	0.75	0.78
		MoC	red0.88	red0.84	0.78	0.74	N/A	0.69	0.76	0.55	0.44
	50/100	OR	red0.93	red0.89	0.74	0.82	N/A	N/A	0.77	0.56	0.67
		WS	red0.95	red0.95	0.86	0.85	N/A	N/A	0.84	0.79	0.65
		MoC	red0.80	red0.79	0.60	0.70	N/A	N/A	0.63	0.59	0.17
IMR90	25/50	OR	red0.97	red0.93	0.80	0.85	N/A	0.57	0.76	0.60	0.4
		WS	red0.98	red0.99	0.93	0.88	N/A	0.75	0.86	0.75	0.47
		MoC	red0.91	red0.95	0.75	0.70	N/A	0.66	0.66	0.53	0.34
	50/100	OR	red0.93	0.89	0.62	0.81	N/A	N/A	0.91	0.56	red0.92
		WS	red0.95	0.94	0.81	0.84	N/A	N/A	red0.95	0.73	0.87
		MoC	red0.81	0.79	0.59	0.69	N/A	N/A	red0.80	0.61	0.54

The top 2 largest values are labeled in red for each line

*OR* overlapping ratio, *WS* weighted similarity, *MoC* measure of concordance

^1^25/50: agreement of 25 kb vs. 50 kb; 50/100: agreement of 50 kb vs. 100 kb

^2^N/A: lack of as least one result

## Discussion

We have demonstrated the usefulness of our proposed algorithm, SuperTAD. In our experiments, SuperTAD outperformed the existing methods in terms of our new metric (overlapping ratio), as well as in robustness and self-consistency.

For further work, we plan to improve SuperTAD in the following aspects. First of all, the complexity of the dynamic programming used in SuperTAD remains relatively high. Some heuristics can be employed to significantly reduce the running time and memory requirement. A relatively difficult obstacle is that the current definition of the coding tree does disallow for the TADs to overlap. A challenging future work is to devise novel strategies that will allow us to identify the overlap between TADs.

## Conclusions

In this article, we proposed SuperTAD, a novel method to find the optimal coding tree with the minimum structural entropy from the Hi-C matrix. A coding tree represents a hierarchical structure of TADs. SuperTAD operates in two different modes in restricting the size of the coding tree, namely by the number of leaves, or by tree height. The first mode, SuperTAD, requires no user-defined parameters, while the second mode, SuperTAD(*h*), requires a parameter *h* to determine the number of layers. Both modes run in polynomial time and find a globally optimal solution to the coding tree problem. In our experiments, SuperTAD performed better than existing methods in our metrics, as well as in robustness and self-consistency. Furthermore, the coding trees computed are proved to be biologically meaningful.

## Methods

### Hi-C data collection

We download the processed Hi-C contacts (.hic format) of two human cell lines (GM12878 and IMR90) from NCBI with accession number GSE63525 [28], and both are in situ Hi-C protocol datasets. GM12878 dataset has 4.9 B contacts while IMR90 dataset has 1.1 B contacts. The contacts are merged across primary and replicates with a filtering MAPQ ≥30. The raw matrix is further normalized by Juicer [36] built-in KR (Knight-Ruiz) normalization as normalized Hi-C matrix (referred to as *KR matrix*).

### ChIP-seq data collection and analysis

To obtain the enrichment information of epigenetics characteristics, we downloaded the Transcription Factor (TF) ChIP-seq from ENCODE (https://www.encodeproject.org/). For structural proteins like CCCTC-binding factor (CTCF), RAD21 and SMC3, we downloaded the optimal IDR thresholded peaks. And for histone modifications H3K27me3 and H3K36me3, we downloaded the fold change over control signals. The experiment accession numbers are summarized in Additional file 1: Table S1.

To assess the enrichment of structural proteins around TAD boundaries, we firstly summed the ChIP-seq peaks into 5-kb intervals around boundaries. Then, we calculated the average peak number of the intervals from two regions, one is the region surrounding the boundaries (the bin detected as boundary and ± 1 bin, referred to as *peak*), the other is the 100-kb region located 400 kb away from the boundaries at both sides (referred to as *background*). The TAD boundaries are defined as the ends of TADs. We computed the fold change between the average peak number of peak and background per TAD and took the average. Zero value of the average fold change stands by no enrichment around boundaries and a higher value means the boundaries are enriched for the structural proteins.

To assess the enrichment of two histone modifications, H3K27me3 (repressing) and H3K36me3 (activating) within TADs, we adopted the modified analysis from the work of Zufferey et al. [27]. We summed the ChIP-seq signals into intervals with fixed length (10% of the resolution). Next, we computed the log10 ratio between H3K27me3 and H3K36me3 for each interval (LR value) and computed the average LR values of intervals within each TAD as the observed LR values. Then, we performed 1000 times shuffling to calculate the empirical *p* value for each TAD and corrected the empirical *p* value through false discovery rate (FDR) using the Benjamini-Hochberg (BH) method. With the constraint that FDR-corrected *p* value ≤0.1, we classified the TADs into two groups, one is enriched for either H3K27me3 or H3K36me3, the other is enriched for neither (FDR-corrected *p* value >0.1). We further divided the former group (FDR-corrected *p* value ≤0.1) into two subgroups, TADs enriched for H3K27me3 and enriched for H3K36me3 based on each TAD’s observed LR value. We reported the fraction of the three clusters. A higher fraction of TADs enriched for either H3K27me3 or H3K36me3 is considered to reflect a more biological meaningful result of the algorithm.

### The SuperTAD algorithm

#### Notation and definition

To study TAD or loops, researchers often partition the genome into a sequence of bins or windows, where a bin contains a fix length segment of the genome. Denote the number of bins as *n*. Denote a Hi-C matrix as *X*={*x*_*i*,*j*_}, where *x*_*i*,*j*_,1≤*i*,*j*≤*n*, is a non-negative real number which represents the interaction frequency between bins *i* and *j*; it is often the normalized read count which hit both bin *i* and *j* simultaneously. *X* is symmetric. The diagonal elements are set to be zeros in the matrix. A symmetric matrix is equivalent to a undirected graph. In deDoc [33], *X* is interpreted as a weighted graph and the objective is to find a hierarchical structure of TADs where the structural information (entropy) is minimum.

#### Coding tree

Structure information theory is proposed to measure the uncertainty embedded in the dynamics of a graph [37]. Finding a partition of the graph with the minimum structural entropy is akin to finding a partition which can best represent the original graph while reducing all the random variation and noise to a minimum. Here, we introduce the definitions in structure information theory that are relevant to our TAD finding problem.

A *coding treeT* of *X* forms a hierarchical partitioning of the bins of the Hi-C matrix. The coding tree can be multi-nary. Each node of the tree contains (or codes) a set of consecutive bins. The root *λ*_*T*_ represents, or codes, the entire genome. Each tree node codes a subset of consecutive bins along the genome. The children of each tree node partition the bins of their parent node. These partitions are used to define TAD boundaries and each node is a TAD candidate.

Denote the bins represented by a node *u*∈*T* as *b*_*T*_(*u*), and denote its volume as *V*(*u*); that is, $V(u)=\sum _{i\in b_{T}(u), j\in b_{T}(\lambda _{T})} x_{i, j}$. The structural entropy of *u* is then defined as

1 $$ S_{T}(X; u) = -\frac{g(u)}{2m}\log_{2}\frac{V(u)}{V({p_{T}(u)})},  $$

where $g(u)=\sum _{i\in b_{T}(u), j\in b_{T}(\lambda _{T})-b_{T}(u)} x_{i, j}, p_{T}(u)$ is the parent node of *u*, and $2m=\sum _{i,j\in b_{T}(\lambda _{T})}{x_{i, j}}$. Clearly, if a node contains only one bin, *g*(*u*)=*V*(*u*). Denote the leaf node in *T* a bin *b*_*i*_ belongs to as *e*_*T*_(*b*_*i*_), let the structural entropy of bin *b*_*i*_ in *T* as

2 $$ S_{T}(X; b_{i}) = -\frac{g(b_{i})}{2m}\log_{2}\frac{V(b_{i})}{V(e_{T}(b_{i}))},  $$

where $g(b_{i})=\sum _{j\ne i} x_{i, j}$, and $V(b_{i})=\sum _{j} x_{i, j}$

According to the definitions of *g* and volume *V*, it is clear that the following hold:

##### **Lemma 1**

If the bins coded by node *v*_1_,..., *v*_*ℓ*_ partition the bins coded by *v*, then $\sum _{1\le i\le \ell } g(v_{i})\ge g(v)$, and $\sum _{1\le i\le \ell } V(v_{i})\ge V(v)$.

We write *p*_*T*_(*u*) as *p*(*u*) and *e*_*T*_(*b*_*i*_) as *e*(*i*) when the context are clear.

The root *λ*_*T*_ has a structural entropy of 0. The structural entropy *S*_*T*_(*X*) of a coding tree is the sum of the structural entropy of all its nodes and all the bins; that is,

3 $$ S_{T}(X)=\sum_{u\in T} S_{T}(X; u)+\sum_{1\le i\le n} S_{T}(X; i)  $$

The *optimal coding tree* is for the matrix *X* a tree *T*_*opt*_(*X*) with minimal structural entropy. The TAD finding task is then to find an optimal coding tree.

#### Finding optimal coding trees

First, we prove the following results:

##### **Lemma 2**

The structural entropy of an optimal coding tree with *k*+1 leaves is always no more than that of an optimal tree with *k* leaves, where *k* is an integer.

##### *Proof*

Assume *T*_*a*_ is a tree of *k* leaves. Without loss of generality, we assume its first leaf *v* contains bins 1 to *ℓ*,*ℓ*≥2. We transform *T*_*a*_ into *T*_*b*_ by: (1) Creating new leaves *v*_1_, and *v*_2_, where *v*_1_ codes bins 1,..., *j*, *j*≤*ℓ* and *v*_2_ codes bins *j*+1,..., *ℓ*, (2) *v*_1_ and *v*_2_ are the children of *v*. We just need to prove that *T*_*b*_ has the same or lower structural entropy than *T*_*a*_.

$$ \begin{aligned} S_{T_{a}}(X)-S_{T_{b}}(X) =& -S_{T_{b}}(X; v_{1})-S_{T_{b}}(X; v_{2})+\sum_{1\le i\le\ell} (S_{T_{a}}(X; i)-S_{T_{b}}(X; i))\\ =& \frac{g(v_{1})-\sum_{1\le i\le j}g(b_{i})}{2m}\log_{2}\frac{V(v_{1})}{V(v)} +\frac{g(v_{2})-\sum_{j< i\le \ell }g(b_{i})}{2m}\log_{2}\frac{V(v_{2})}{V(v)}\\ \ge &0. \end{aligned}  $$

Therefore, $S_{T_{a}}(X)\ge S_{T_{b}}(X)$. The optimal tree with *k*+1 leaves will have an entropy no more than $S_{T_{b}}(X)$. Hence, our statement holds. □

Clearly, due to Lemma 2, we need to restrict the number of leaves to have an optimal coding tree. We assume the number of leaves in the coding tree is *k*.

Without loss of generality, we assume that each internal node *u* has at least two children. If a node *u* is the only child of its parent *p*(*u*), then *V*_*T*_(*u*)=*V*_*T*_(*p*(*u*)), and *S*_*T*_(*X*;*u*)=0, showing *u* to be redundant.

We next show that an optimal tree can be required to be binary without loss of generality.

##### **Lemma 3**

For every contact matrix *M*, there exists a binary coding tree of minimum structural entropy.

##### *Proof*

**Fig. 4:**
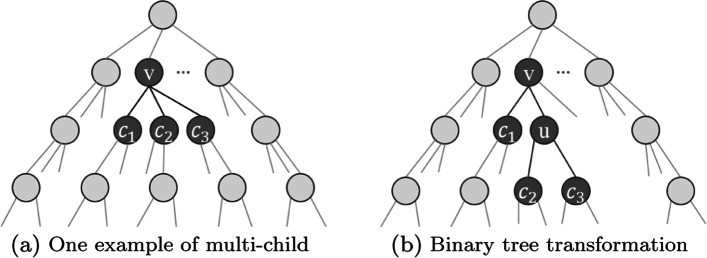
Two kinds of structure for the identical node {*v*,*c*_1_,*c*_2_,*c*_3_}. **a** A sub-structure embedded in the whole tree that node *v* has multiple children, node {*c*_1_,*c*_2_,*c*_3_}. **b** The binary transformation of **a** structure that node *c*_2_ and *c*_3_ firstly merged as node *u* then node *u* becomes the new child of node *v*. It turns out the binary structure **b** always has the smaller structural entropy

Given a node *v* in a tree *T*_*a*_ with more than two children, *c*_1_,*c*_2_,...,*c*_*ℓ*_,*ℓ*≥3, we can transform optimal coding tree *T*_*a*_ into *T*_*b*_ such that, (1) in *T*_*b*_, *v* has children *c*_1_ and *u*, *u* has children *c*_2_,...,*c*_*ℓ*_; and (2) all the other parts of *T*_*a*_ and *T*_*b*_ are the same (see Fig. 4). We just need to prove that *T*_*b*_ has no more structural entropy than *T*_*a*_.

4 $$ \begin{aligned} S_{T_{a}}(X)-S_{T_{b}}(X) & = -S_{T_{b}}(X; u)+\sum_{2\le i\le\ell}S_{T_{a}}(X; c_{i})- \sum_{2\le i\le\ell}S_{T_{b}}(X; c_{i})\\ & = \frac{g(u)-\sum_{2\le i\le \ell} g(c_{i})}{2m}\log_{2}\frac{V(u)}{V(v)}\\ &\ge 0. \end{aligned}  $$

Therefore, the statement holds. □

Note that both Lemmas 2 and 3 hold for general graphs. To find an optimal coding tree, we merely need to search the binary trees. Here, we adopted a dynamic programming approach to find the tree. Let *S*(*i*:*j*,*k*) be the structural entropy of the optimal binary coding tree when partitioning bins (*b*_*i*_,*b*_*i*+1_,...,*b*_*j*_) with *k* leaves, denote *H*_*l*_(*i*_*l*_:*i*_*r*_,*j*) as structural entropy of the node containing bins $\left \{b_{i_{l}},b_{i_{l}+1},..., b_{j}\right \}$ with its parent containing bins $\left \{b_{i_{l}}, b_{i_{l}+1},..., b_{i_{r}}\right \}, i_{l}\le j \le i_{r}$; and denote *H*_*r*_(*i*_*l*_:*i*_*r*_,*j*) as structural entropy of the node containing bins $\left \{b_{j+1},b_{j+2},..., b_{i_{r}}\right \}$ with its parent containing bins $\left \{b_{i_{l}}, b_{i_{l}+1},..., b_{i_{r}}\right \}, i_{l}\le j\le i_{r}$.

Then, we can write the recurrent relations to find the optimal binary coding tree with *k* leaf nodes for *X*

5 $$  S(1:n, k) = \min_{1\le i < n, 1\le k_{1} < k}\left\{S(1:i, k_{1}) + S(i+1:n, k-k_{1}) + H_{l}(1:n, i) + H_{r}(1:n, i)\right\}  $$

where *S*(1:*n*,*k*) is the structural entropy of the optimal binary coding tree with partitioning bins {*b*_1_,*b*_2_,...,*b*_*n*_} with *k* leaves. *k*_1_ and *k*−*k*_1_ are the number of leaves in the left subtree and right subtree, respectively (Additional file 1: Figure S2).

There are *O*(*n*^3^) possible *H*_*l*_(1:*n*,*j*) and *O*(*n*^3^) possible *H*_*r*_(1:*n*,*j*) terms, each can be calculated in *O*(1) time. Hence, *O*(*n*^3^) time is necessary to compute these *H* terms. A table of size *O*(*kn*^2^) can be created to store the values of *S*(1:*n*,*k*), and each value of *S*(1:*n*,*k*) takes time *O*(*kn*). Hence,

##### **Theorem 1**

There exists an algorithm that finds the optimal coding tree of *k* leaves with time complexity *O*(*k*^2^*n*^3^).

Also, we may restrict the height of the coding tree. A heuristic algorithm exists for the problem [33]. Here, we solve the problem exactly by a dynamic programming. We propose SuperTAD(*h*) which restricts the size of the coding tree by assuming the optimal coding tree is to be of height at most *h*. The tree may not be binary and a node can have more than two children. Our dynamic programming is as follows. Let *T*(*l*:*r*,*p*,*k*,*h*) store the structural entropy of a multi-nary optimal coding tree where (1) the root codes bins {*b*_*l*_,*b*_*l*+1_,...,*b*_*r*_}; (2) children nodes partition bins {*b*_*l*_,*b*_*l*+1_,...,*b*_*p*_},*p*≤*r*; (3) there are a total of *k* leaves; and (4) the height is at most *h*. Then, we can write the recurrence relation as follows:

6 $$ \begin{aligned} T(l:r, p, k, h)=&\min_{l\le i < p, 1\le k_{1} < k}\{ \min\{T(l:r, i, k_{1}, h), T(l:i, i, k_{1}, h-1)+H_{l}(l:r, i)\}\\ &+ T(i+1:p, p, k-k_{1}, h-1) + H(l:r,i+1:p)\} \end{aligned}  $$

where *H*(*l*:*r*,*i*+1:*p*) is the structural entropy of a node which codes the bins {*b*_*i*+1_,*b*_*i*+2_,...,*b*_*p*_}, where its parent node codes bins {*b*_*l*_,*b*_*l*+1_,...,*b*_*r*_} (Additional file 1: Figure S3).

##### **Theorem 2**

There exists an algorithm that finds the optimal coding tree of at most height *h* and at most *k* leaves with time complexity *O*(*n*^4^*k*^2^*h*).

The time complexity can be reduced by extracting a candidate set of TAD boundaries prior to applying the algorithm. This shows that *the optimal coding tree with restricted height problem is polynomial-time solvable*.

#### Determine the number *k* of leaves

As mentioned, structural entropy decreases with an increase of the number of leave nodes *k*. We consider the problem of determining a suitable *k*. First, we propose a Bayesian Information Criteria (BIC) approach. Second, we normalize the elbow point at the structural entropy vs. *k* curves. Third, we try to compare the structural entropy to a background model; that is, we try to derive the structural entropy in the ideal contact matrix and use it as a normalization factor. A fourth approach is based on our observation that as *k* increases, the sum of the structural entropy of the leaf nodes drops at first, but increases after a minimum is reached. We explore using the *k* value which corresponds to this minimal entropy for the leaves.

#### Filter TADs

Each node in a coding tree gives a potential candidate for defining TADs. We consider the task of filtering out the nodes which are unlikely to be TAD. A node is likely to be a TAD if the intra-interactions are much dense. To eliminate the influence of hierarchy and compute the inherent density for each TAD, we compute the average interaction frequency at three layers: the parent node, the node’s children, and the node itself. Starting from the root, we iteratively deduct the influence of the parent and children for each node up to the leaf nodes. In this way, we calculate each node’s inherent density from the top to the bottom of the coding tree.

Based on the empirical distributions of contact frequencies in the Hi-C matrix, the contact frequency decreases with the increase of distance. Next, we cluster all the nodes into two sets based on their inherent density and size, repeating with 1000 times random initialization. We select the set of TADs that shows a strong negative relationship between their sizes and inherent density. We calculate the probability of being selected for each TAD candidate. The candidates that show low probability, lower structure entropy than their parent and close to equally split from their parent are discarded (Additional file 1: “More details in nodes filtering” section, Figure S4, S5).

### Assess the similarity between two coding trees

Given two coding trees *X* and *Y* of the same Hi-C matrix. Suppose that *X*={*x*_1_,*x*_2_,..,*x*_*m*_} and *Y*={*y*_1_,*y*_2_,...,*y*_*n*_} where each node *X*_*i*_ or *Y*_*j*_ is a consecutive set of bins. We consider evaluating the similarity between *X* and *Y*.

The work of deDoc [33] proposes the *weighted similarity* between *X* and *Y*, which is defined as $ws_{X}^{Y}$

7 $$ ws_{X}^{Y} = \frac{\sum_{j=1}^{n} |y_{j}|\cdot S_{X}^{Y}(j)}{\sum_{j=1}^{n} |y_{j}|}  $$

8 $$ S_{X}^{Y}(j) = \max_{i=1}^{m} \left\{ \frac{|x_{i} \cap y_{j}|}{\sqrt{|x_{i}|\cdot |y_{j}|}} \right\}  $$

However, the definition shows that weighted similarity is an asymmetry metric, and it is hard to determine the similarity when there is a big difference between $ws_{X}^{Y}$ and $ws_{Y}^{X}$.

Zufferey et al. [27] adopted the measure of concordance (*MoC*), a symmetric metric to compare clustering assignments, which is defined as *MoC*(*X*,*Y*)

9 $$ MoC(X, Y) = \left\{ \begin{aligned} 1,\ if\ N_{X}=N_{Y}=1 \\ \frac{1}{(\sqrt{N_{X} N_{Y}}-1)}\left(\sum_{i=1}^{N_{X}}\sum_{j=1}^{N_{Y}} \frac{\left\|F_{i,j}\right\|^{2}}{\left\|X_{i}\right\|\left\|Y_{j}\right\|} -1\right),\ \text{otherwise}\\ \end{aligned} \right.  $$

However, the MoC is upper and lower bounded only if the partitions are disjoint (any two TADs of *X* or *Y* do not have overlap). To adopt the MoC for assessing the agreement of two hierarchical TAD structures, we only selected the level one of hierarchy (the TAD can no further partition) and added the inter-TAD regions into the assessment as [27].

In this work, we use, in conjunction with the weighted similarity and MoC, a new symmetry metric we call *overlapping ratio* to measure coding trees similarity. First, we build a Bipartite Graph *G*=(*V*,*E*) in which the vertex set can be partitioned *V*={*X*,*Y*}, and every edge *e*∈*E* links one node in *X* and the other node in *Y*. We define the weight of edges as the intersection between the two linked nodes, denoted as *w*(*x*_*i*_;*y*_*j*_). Obviously, the graph *G* is complete.

Then, we apply Maximum Bipartite Matching to the graph with the goal of finding a maximum matching *M* that the summation of selected edges’ weight is maximum. That is, we find the global optimal matching for every node in *X* and *Y*. The overlapping ratio between *X* and *Y* is defined as the function *S*(*X*,*Y*)

10 $$ S(X, Y) = \frac{\sum_{i=1}^{M} w\prime\left(x_{i};*\right)+ \sum_{j=1}^{N} w\prime\left(*; y_{j}\right)}{\sum_{i=1}^{M} |x_{i}|+\sum_{j=1}^{N} |y_{j}|}  $$

where *w*′ is the edge weight in the maximum matching *M* and *w*′(*x*_*i*_;∗) is defined as

11 $$ w\prime(x_{i};*) = \left\{ \begin{aligned} w(x_{i}, y_{j}), \text{if edge} ~e\left(x_{i}; y_{j}\right) ~\text{is selected in }{M} \\ 0, \text{none of} ~e\left(x_{i}; *\right) ~\text{is selected in }{M}\\ \end{aligned} \right.  $$

The overlapping ratio is symmetric, *S*(*X*,*Y*)=*S*(*Y*,*X*). The value of overlapping ratio between any coding trees ranges from 0 to 1, where 1 indicates that the two coding trees are the same while 0 indicates the two coding trees contain no intersection between any pair of *x*_*i*_ and *y*_*j*_.

### The SuperTAD C++ package

SuperTAD is implemented as a command line tool in C++. We compiled and tested our software on both local computers and a Linux server with CentOS 7.6 pre-installed that has 96 12-core processors and 598 GB memory. Our method and software guarantee accuracy while do not sacrifice computational performance. The source codes of SuperTAD package are available at https://supertad.deepomics.org/, where the example dataset is also deposited. The version used in the manuscript is permanently available at 10.5281/zenodo.4314123.

## Supplementary Information

**Additional file 1** Supplementary Information.

Additiona file 2 **Figure S6**. The robustness comparison between SuperTAD and deDoc(E) under various noise ratios and sizes. **a** The influence of noise on the performance of both methods. The *x*-axis indicates the increase in noise ratio from 5 to 50% by 5%, while the boxes show the value distribution of certain metrics among 100 repeated experiments. **b** The influence of variance in TAD size (length) on the performances of both methods. The *x*-axis indicates the increase in standard deviation of TAD size while the boxes show the value distribution of certain metrics among 100 repeated experiments. The boxes of SuperTAD are colored in blue while deDoc are in orange. The colored line links the mean value (the green point in boxes) across boxes. For boxplots, centerline indicates the median, box limits indicate upper and lower quantiles, whiskers indicate the 1.5 interquantile range, and points indicate outliers.

Additiona file 3 **Figure S7**. Comparison between SuperTAD(2) and deDoc using real Hi-C matrix for in situ Hi-C GM12878 and IMR90 cell lines. We first apply SuperTAD(2) and deDoc on KR (normalized) Hi-C matrix of two human cell lines (GM12878 and IMR90) at 25-kb bin resolution. The boxplots show the statistics on **a** length, **b** structural entropy, and **c** contact density of inferred TADs for both methods. The box shows the value distribution of each method for each cell line (blue boxes represent SuperTAD(2) while orange boxes represent deDoc). The marked numbers and dashed lines in red both indicate the mean value for each box. The contact density is defined as the number of intra-TAD contacts divided by TAD length. **d** The structure entropy of the coding tree detected by SuperTAD(2) and deDoc for both cell lines at 25-kb and 50-kb resolutions. **e** The fold change of structural proteins peak number (CTCF, RAD21, SMC3) between peaks (regions around boundaries) and background (regions located 400 kb away from the boundaries). The higher value indicates more enrichment of structural proteins around boundaries. **f** The cummulative bar diagram shows the fraction of TADs from three groups: enriched for H3K27me3 (FDR-corrected *p* value ≤0.1, the blue bar); enriched for H3K36me3 (FDR-corrected *p* value ≤0.1, the orange bar); no significant enrichment (FDR-corrected *p* value >0.1, the green bar). **g**, **h** The heatmap and inferred boundaries with various inputs for GM12878 and IMR90 cell lines. Each heatmap exhibits different results with two inputs. Text in the upper/lower triangle indicates the input matrix’s information, and the plotted boundaries on the same side present the corresponding result. The similarity between boundaries in different colors shows the robustness of performance between 25-kb and 50-kb bin resolution (or raw and KR) matrix for each method. Note that the heatmap is asymmetric when comparing two results from raw and KR matrices.

Additiona file 4 **Figure S8**. Consistency comparison for the same cell line with 25-kb vs. 50-kb resolutions among all the methods. The heatmap and detected boundaries with 25-kb and 50-kb bin resolution input for GM12878 (the top line) and IMR90 (the bottom line) cell lines. The detected domains from 25-kb resolution are colored in blue at the upper triangle, and 50-kb resolution results are in pink at the lower triangle (as the texts indicate). The similarity between boundaries in different colors shows the robustness of performance between 25-kb and 50-kb bin resolution matrices for each method.

**Additional file 5** Review history.

## Data Availability

SuperTAD is available at https://github.com/deepomicslab/SuperTAD and https://supertad.deepomics.org/[38], under MIT license. The versions used in the manuscript are permanently available at 10.5281/zenodo.4314123 [39]. Hi-C data: The processed Hi-C contacts (.hic format) of two human cell lines, GM12878 and IMR90, are downloaded from Rao et al. [28] (GEO accession number: GSE63525), and both were in situ Hi-C protocol datasets. The raw and KR normalized Hi-C contact maps at 25-kb, 50-kb, and 100-kb resolutions are included in this study. Epigenomic data: The Transcription Factor (TF) ChIP-seq data of CTCF, cohesin protein complex RAD21, and SMC3 were downloaded from ENCODE project (https://www.encodeproject.org/). The optimal IDR thresholded peaks were downloaded in bigBed format. The Histone ChIP-seq data of H3K27me3 and H3K36me3 were also downloaded from the ENCODE project. The fold change over control signals was downloaded in bigWig format.
