# Supplementary material for: SuperTAD: robust detection of hierarchical topologically associated domains with optimized structural information
Source: Genome Biol. 2021 Jan 25;22:45. doi: 10.1186/s13059-020-02234-6 (PMC7831269; doi:10.1186/s13059-020-02234-6)
Supplement: Supplementary file 1 — Additional file 1 Supplementary Information. [file 13059_2020_2234_MOESM1_ESM.pdf]

Supplementary Information

SuperTAD: Robust detection of hierarchical topologically associated domains with the optimized structural information

*Yuwei Zhang, Mengbo Wang and Shuaicheng Li**

Department of Computer Science, City University of Hong Kong, Kowloon

High consistency of SuperTAD results across 50kb vs. 100kb resolutions

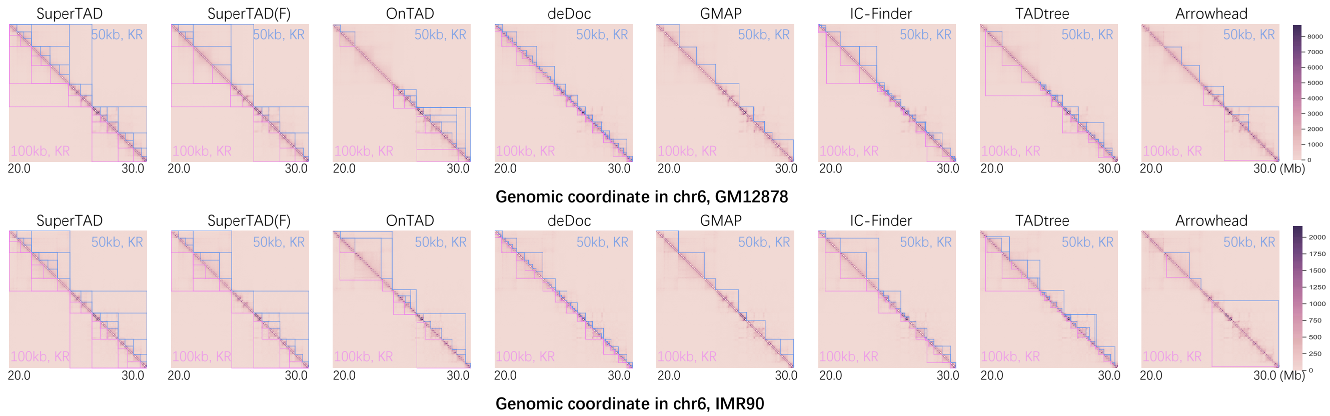

Figure S1. The heatmap and predicted boundaries with 50kb and 100kb bin resolution input for GM12878 (the top line) and IMR90 (the bottom line) cell lines. The predicted domains from 50kb resolution are colored in blue at the upper triangle, and results of 100kb resolution are in pink at the lower triangle (as the texts indicate). The similarity between boundaries in different colors shows the robustness of performance between 50kb and 100kb bin resolution matrices for each method. No boundaries are shown on the lower triangle of GMAP as GMAP fails to detect TADs from the identical input at 100kb resolution for both cell lines. The result of 3DNetMod is omitted here as 3DNetMod fails to detect TADs from the identical input at 50kb and 100kb resolutions for both cell lines.

Remarks on the recurrent relations of dynamic programming

We provide two modes of SuperTAD for users and both are to find the eligible coding tree with global minimum structure entropy. For each mode, we propose a recurrent relation of dynamic programming (as the formula (5)(6) in the paper), and we will explain them in detail in this section.

The first recurrent function is as follows, which is to find the binary coding tree with minimum structure entropy.

$$S(1:n,k) = \min_{1\leq i<n, 1\leq k_1<k} \{S(1:i,k_1) + S(i+1:n,k-k_1) + H_l(1:n,i) + H_r(1:n,i)\}$$

When calculating each value in S, the value of i and k are also stored. When performing backtracing, we start from the state $S(1:n,k)$, obtain the optimal splitting position i and divide the current node into two nodes. We keep backtracing until $k=1$, which means the current node can no longer split. It is the same way to build a binary tree from root to the leaves. Since the bin is continuous in real Hi-C matrix, we store the start position and end position to represent the range of bin set and the value of k for each node. The value of k stands by the number of leaves contained in the

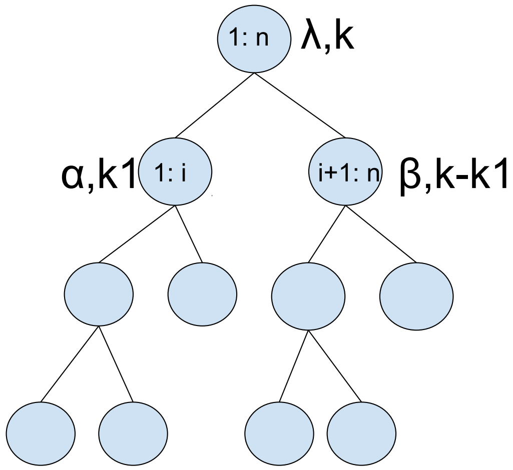

Figure S2. An example of binary coding tree. The top node is root λ and k stands by the leaves number. The node α is the left children of root λ and k_1 represents the leaves number of left sub-structure (node α as root). Likewise, The node β is the right children of root λ and k_2 represents the leaves number of the right sub-structure (node β as root).

sub-tree structure where the current node as root. As shown in fig S2, the root node λ is associated with the whole bin set $\{b_1, b_2, ..., b_n\}$ and k leaves. In the first step of backtracing, the root λ splits into node α and β. The bin set of node α is $\{b_1, b_2, ..., b_i\}$. $k1$ stands by the leaves number of the left sub-structure (node α as root). The binary coding tree is unbalanced, and the height will increase when the leaf nodes split. So the height of binary coding tree ranges from $\lceil \log_2 k \rceil$ to $k-1$, where k is the number of leaves and the height of tree that only contains root is assumed as zero.

As for the second mode, we constrain the tree size by height . Users can manually select the value of parameter h to determine the height of root node λ. The recurrent function of dynamic programming as follows

$$T(1:n, n, k, h) = \min_{1 \le i < n, 1 \le k_1 < k} \{\min\{T(1:n, i, k_1, h), T(1:i, i, k_1, h-1) + H(1:n, 1:i)\} + T(i+1:n, n, k-k_1, h-1) + H(1:n, i+1:n)\}$$

The recurrent function is different from formula (6) in the manuscript, the latter is more general. As shown in fig S3, the root λ is associated with the whole bin set $\{b_1, b_2, ..., b_n\}$ and k leaves, h stands by the height. The first step of backtracing starts from λ and determines the rightmost children β, leaving the rest (grey box) for next backtracing. As the node in a multi-nary tree can have an arbitrary number of children, the grey box can have one or more nodes at the same height of β. If $T(1:n, i, k_1, h)$ is chosen through the inner min comparing, then the number of nodes in the grey box remains unknown and h also remains unchanged. Otherwise, there is only one node in the grey box, $H(1:n, 1:i)$ represents the structure entropy of the only node. Note that the value of h only decreases by one when one certain child has been determined, and the range of parent node will also change accordingly. We keep splitting until the value of k equals to one and h equals to zero (the node is a leaf node).

More details in nodes filtering

When applying nodes filtering, we first calculate the inherent density of each node in the optimal coding tree. We assume the density of TAD (here stands by the average intra-interaction frequency in the TAD) directly obtained from the input matrix is determined by the parent, the children, and the node itself. Therefore, we start from the root and iteratively subtract the influence of parent and children to get the node's inherent density.

Based on the empirical distributions of contact frequencies in the Hi-C matrix, the contact frequency will decrease with the increase of distance. Therefore we assume a linear relationship between the contact frequency and distance of any

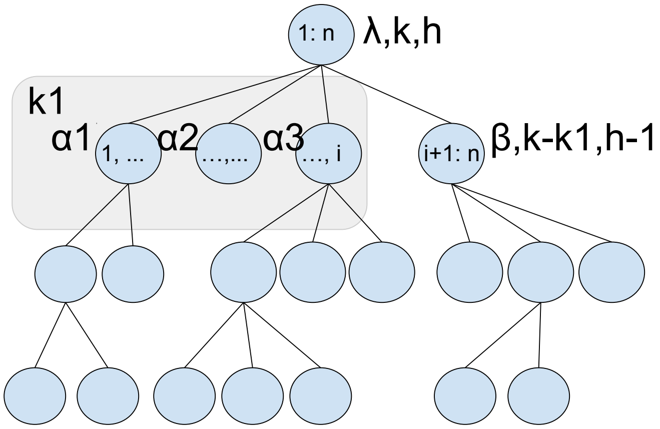

Figure S3. An example of a multi-way coding tree. The top node is root λ, k stands by the leaves number, and h is the height of root. The node β is the rightmost children of root λ and the right sub-structure (node β as root) has $k - k_1$ leaves. $h-1$ is the height of node β. For the rest (grey box), the leaves number of the whole structure is k_1, and h remains unchanged. The structure in the grey box will be determined in further backtracing.

two loci i and j, that is $c_{ij} = a \times |i-j| + b$ where $a < 0$. For a node in the coding tree, the equation transforms to depict the relationship between the inherent density and size. We fit all the nodes into two linear equations by iteratively applying the least square method until convergence. To eliminate the influence of random initialization, we repeat 1000 times and compute the probability of being selected for each node (we select the class that follows a stronger negative relationship). We filter out the nodes that satisfy the following rules:

1. The Z-score normalized probability is less than 0.
2. The structure entropy is less than its parent's.
3. The node is close to splitting its parent equally.

We add the third item as nodes tend to equally split from their parent to obtain a lower structure entropy. We simulate the adjacent matrix with two clusters to show the tendency. We consider the bin count N as 10, 50, 100, 200, and 500. For each N, we enumerate all the partitions ($[1, i]$ and $[i+1, N]$) and simulate the adjacent matrix, where the probability of intra-interaction is 0.6, and that of inter-interaction is 0.2. That is, we simulate $N-1$ adjacent matrixes for each N. Then we find the optimal partition position i with minimum structure entropy and record the difference between one child's size and the geometric mean of two children. We draw the boxplot (Fig S4) and find the mean and median of each box is much lower than the expected value, which implies that minimizing structure entropy tends to split node equally. So we use a threshold to separate the nodes that are close to split their parent equally. Given the mean value and bin count, we set the threshold to $\%4$ of bin count.

As shown in fig S5, we can find that the most TADs to be filtered are from level 1 or 2, i.e., the TADs embed no further nesting (level 1) or embed at least one TAD at level 1 (level 2). Fig 5(b) also shows an example that one TAD (bins from [316, 400]) at level 3 is discarded. We provide the filter as an option to the optimal coding tree of SuperTAD.

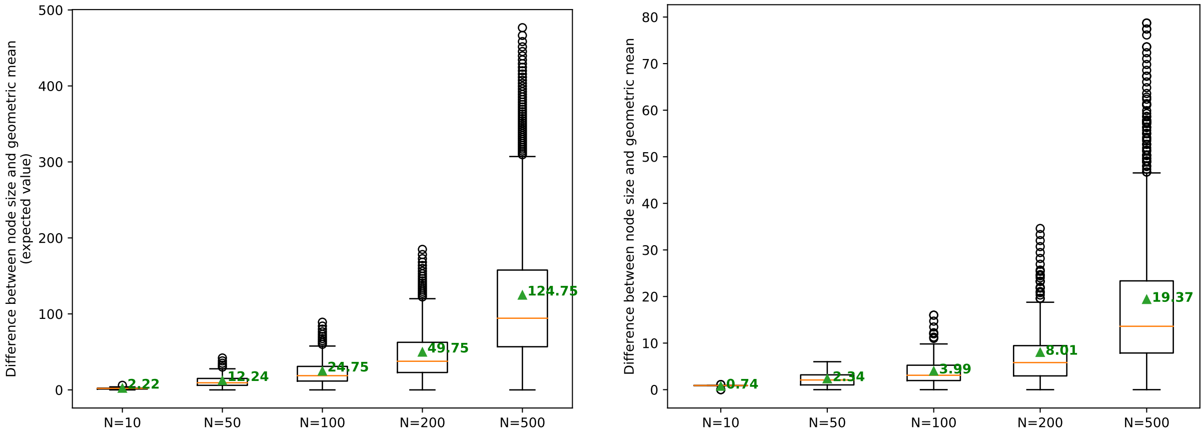

(a) expected difference

(b) observed difference

Figure S4. The comparison between the distribution of expected differences and observed differences. We test the bin count as 10, 50, 100, 200, and 500. For boxplots, orange centerline indicates the median, green triangle as well as annotated number indicate the mean, box limits indicate upper and lower quantiles, whiskers indicate the 1.5 interquantile range and points indicate outliers. The observed value is much lower than the expected value, which increases the unreliability of the nodes that tend to split from their parent equally.

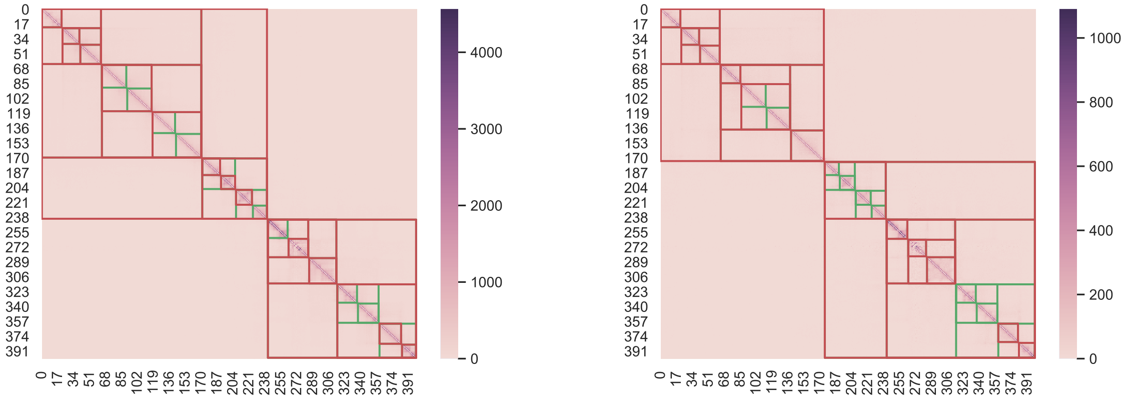

(a) selected TADs in GM12878 cells

(b) selected TADs in IMR90 cells

Figure S5. The comparison between the results before and after nodes filtering. The heatmap shows the selected TADs after filtering compared to all TAD candidates for chr6: 20.0-30.0Mb in **(a)** GM12878 and **(b)** IMR90 cell lines. All the boundaries indicate all the TAD candidates, and the boundaries in red show the selected TADs after filtering while those in green represent the TADs filtered out.

Accession numbers of ChIP-seq experiments

Table S1. Accession numbers of ChIP-seq experiments derived on GM12878 and IMR90

GM12878	CTCF	ENCSR000DKV, ENCSR000DZN, ENCSR000AKB, ENCSR000DRZ[1]
	RAD21	ENCSR000EAC, ENCSR000BMY[1]
	SMC3	ENCSR000DZP
	H3K27me3	ENCSR000DRX
	H3K36me3	ENCSR000DRW
IMR90	CTCF	ENCSR000EFI
	RAD21	ENCSR000EFJ
	SMC3	ENCSR000HPG
	H3K27me3	ENCSR431UUY
	H3K36me3	ENCSR437ORF

[1] The intersection of peaks are obtained through BEDTools.
