# Supplementary figures and images for: SuperTAD: robust detection of hierarchical topologically associated domains with optimized structural information
Source: Genome Biol. 2021 Jan 25;22:45. doi: 10.1186/s13059-020-02234-6 (PMC7831269; doi:10.1186/s13059-020-02234-6)

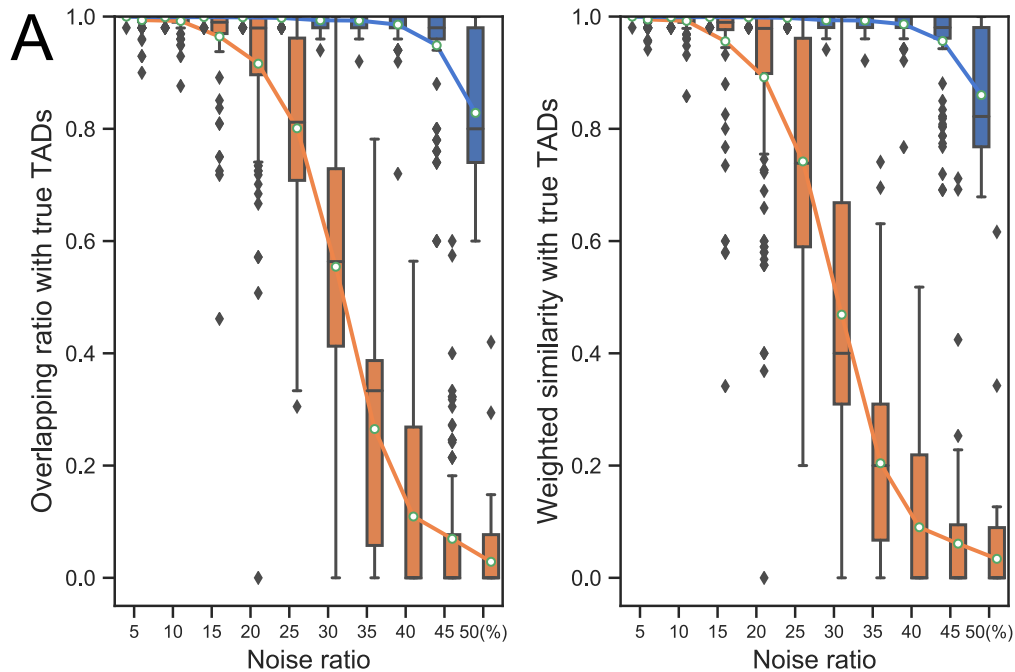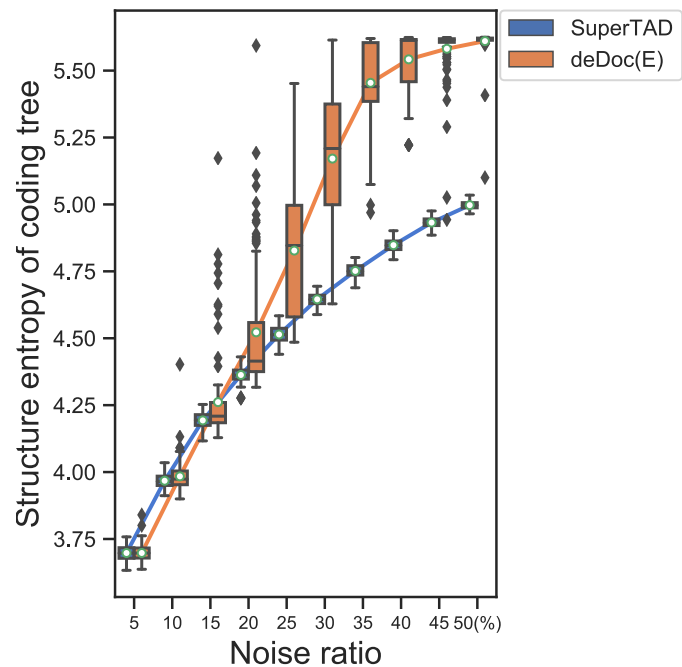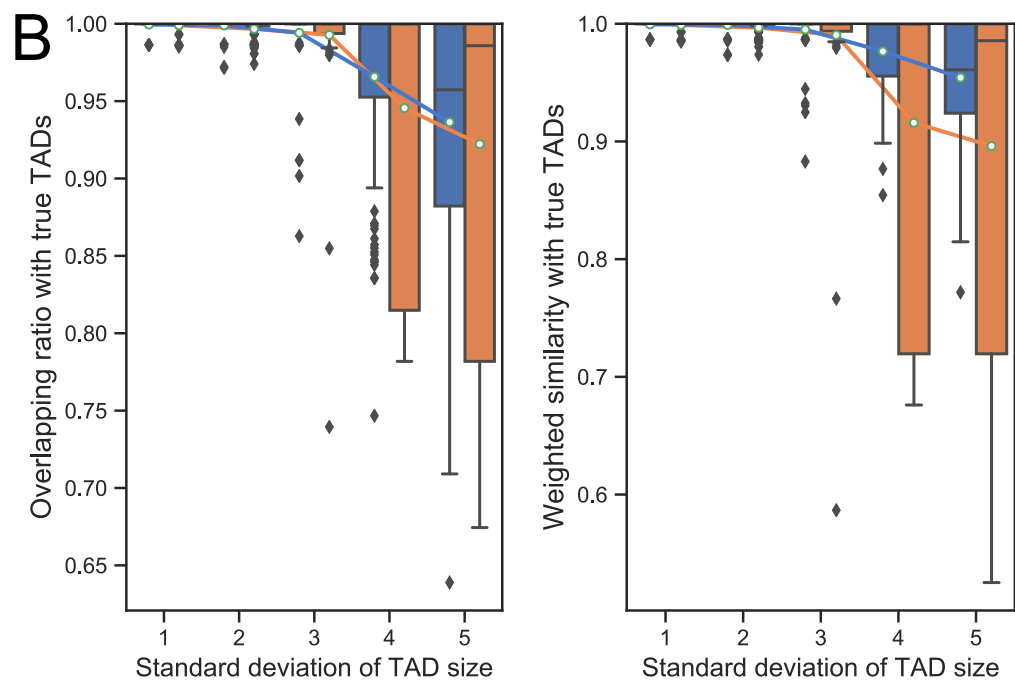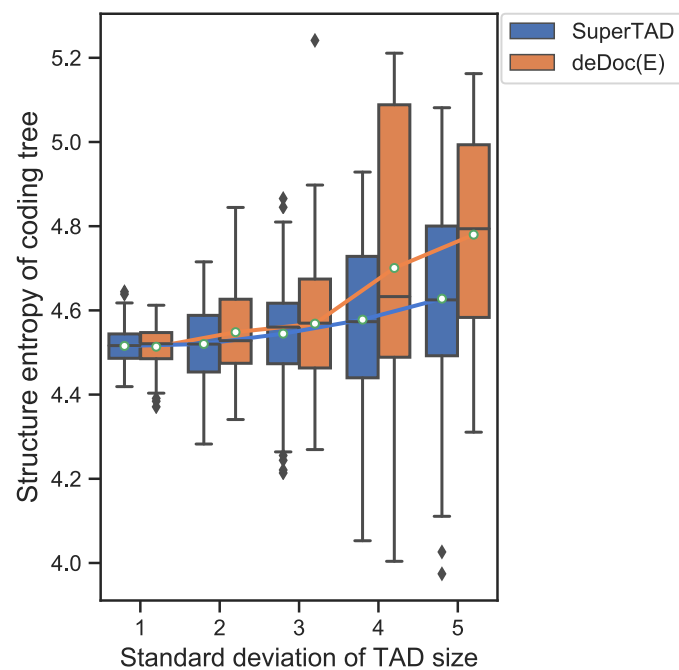

Supplement: Supplementary file 2 — Figure S6. The robustness comparison between SuperTAD and deDoc(E) under various noise ratios and sizes. a The influence of noise on the performance of both methods. The x-axis indicates the increase in noise ratio from 5 to 50% by 5%, while the boxes show the value distribution of certain metrics among 100 repeated experiments. b The influence of variance in TAD size (length) on the performances of both methods. The x-axis indicates the increase in standard deviation of TAD size while the boxes show the value distribution of certain metrics among 100 repeated experiments. The boxes of SuperTAD are colored in blue while deDoc are in orange. The colored line links the mean value (the green point in boxes) across boxes. For boxplots, centerline indicates the median, box limits indicate upper and lower quantiles, whiskers indicate the 1.5 interquantile range, and points indicate outliers. [file 13059_2020_2234_MOESM2_ESM.pdf]

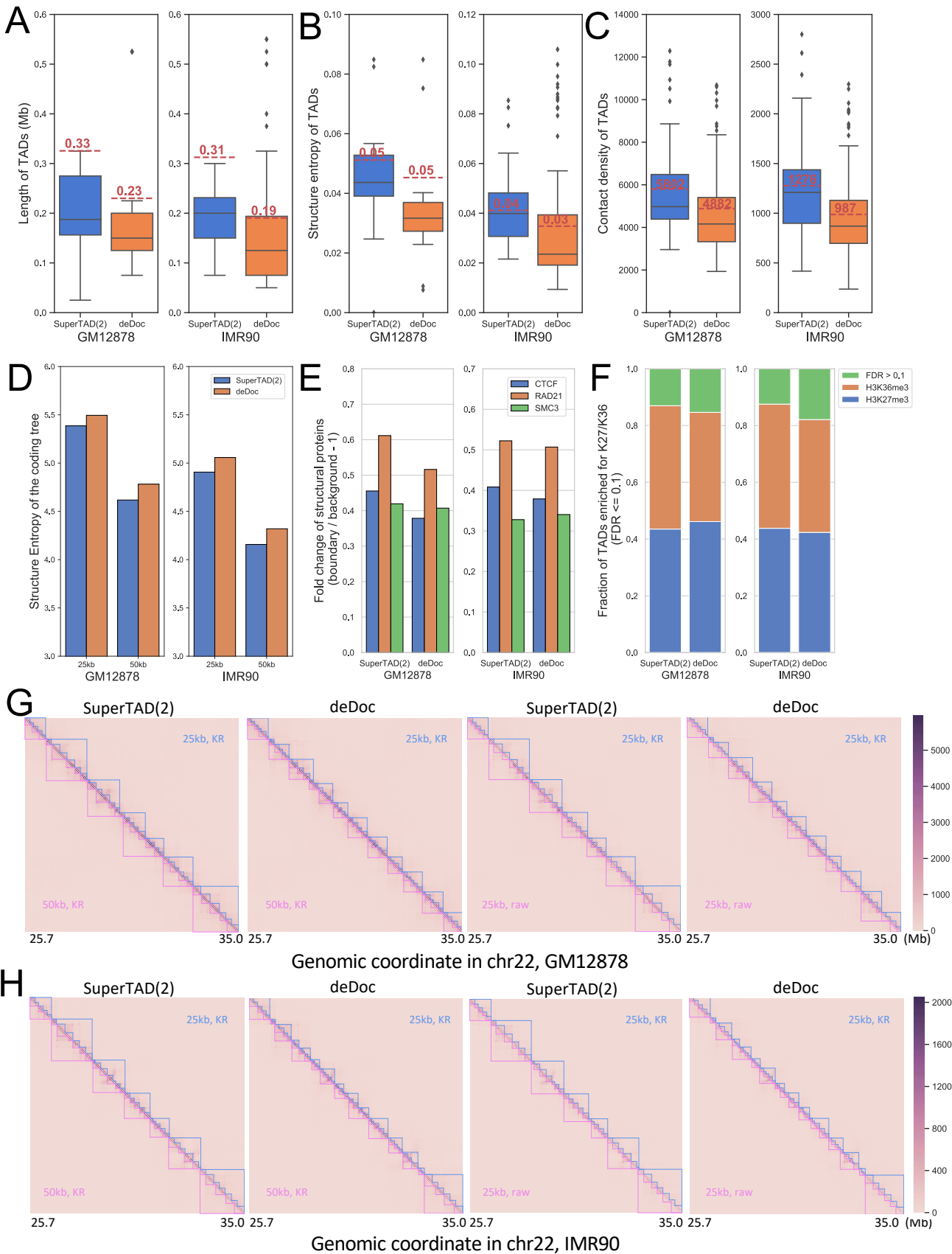

Supplement: Supplementary file 3 — Figure S7. Comparison between SuperTAD(2) and deDoc using real Hi-C matrix for in situ Hi-C GM12878 and IMR90 cell lines. We first apply SuperTAD(2) and deDoc on KR (normalized) Hi-C matrix of two human cell lines (GM12878 and IMR90) at 25-kb bin resolution. The boxplots show the statistics on a length, b structural entropy, and c contact density of inferred TADs for both methods. The box shows the value distribution of each method for each cell line (blue boxes represent SuperTAD(2) while orange boxes represent deDoc). The marked numbers and dashed lines in red both indicate the mean value for each box. The contact density is defined as the number of intra-TAD contacts divided by TAD length. d The structure entropy of the coding tree detected by SuperTAD(2) and deDoc for both cell lines at 25-kb and 50-kb resolutions. e The fold change of structural proteins peak number (CTCF, RAD21, SMC3) between peaks (regions around boundaries) and background (regions located 400 kb away from the boundaries). The higher value indicates more enrichment of structural proteins around boundaries. f The cummulative bar diagram shows the fraction of TADs from three groups: enriched for H3K27me3 (FDR-corrected p value ≤0.1, the blue bar); enriched for H3K36me3 (FDR-corrected p value ≤0.1, the orange bar); no significant enrichment (FDR-corrected p value >0.1, the green bar). g, h The heatmap and inferred boundaries with various inputs for GM12878 and IMR90 cell lines. Each heatmap exhibits different results with two inputs. Text in the upper/lower triangle indicates the input matrix’s information, and the plotted boundaries on the same side present the corresponding result. The similarity between boundaries in different colors shows the robustness of performance between 25-kb and 50-kb bin resolution (or raw and KR) matrix for each method. Note that the heatmap is asymmetric when comparing two results from raw and KR matrices. [file 13059_2020_2234_MOESM3_ESM.pdf]

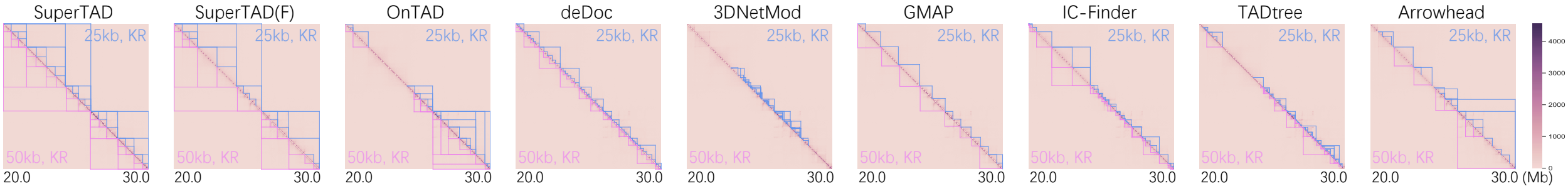

**Genomic coordinate in chr6, GM12878**

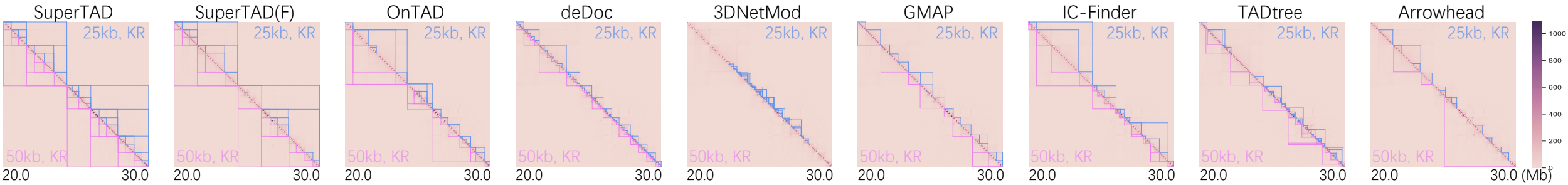

**Genomic coordinate in chr6, IMR90**

Supplement: Supplementary file 4 — Figure S8. Consistency comparison for the same cell line with 25-kb vs. 50-kb resolutions among all the methods. The heatmap and detected boundaries with 25-kb and 50-kb bin resolution input for GM12878 (the top line) and IMR90 (the bottom line) cell lines. The detected domains from 25-kb resolution are colored in blue at the upper triangle, and 50-kb resolution results are in pink at the lower triangle (as the texts indicate). The similarity between boundaries in different colors shows the robustness of performance between 25-kb and 50-kb bin resolution matrices for each method. [file 13059_2020_2234_MOESM4_ESM.pdf]
