# Supplementary material for: SuperTAD: robust detection of hierarchical topologically associated domains with optimized structural information
Source: Genome Biol. 2021 Jan 25;22:45. doi: 10.1186/s13059-020-02234-6 (PMC7831269; doi:10.1186/s13059-020-02234-6)
Supplement: Supplementary file 5 — Additional file 5 Review history. [file 13059_2020_2234_MOESM5_ESM.docx]

Review History

**First round of review**

**Reviewer 1**

**Were you able to assess all statistics in the manuscript, including the appropriateness of statistical tests used?**

Yes: No statistical tests to determine significance between data sets / approaches were used in the manuscript.

**Were you able to directly test the methods?**

No

**Comments to author:**

SuperTAD: Robust detection of hierarchical topologically associated domains with the optimized structural information

Zhang et al.

In this manuscript, Zhang and colleagues introduce the SuperTAD tool to identify TADs and hierarchies ("TADs-within-TADs") using a structural information theory approach.

In recent years, (hierarchical) TAD calling has become a hotly debated topic, with many different approaches having been presented (based on hierarchical clustering, Gaussian mixtures, dynamic programming, graph-theory, structural information theory, etc). The "deDoc" tool (Li et al, Nature Communications 2018) previously used a simplified structural information theory approach to predict TAD hierarchies. In this manuscript, the new tool "SuperTAD" is presented an benchmarked, which is a further optimization of the structural information theory approach.

Based on the information provided in the manuscript, SuperTAD outperforms existing tools (mostly compared to deDoc), although the advance may be somewhat incremental. Moreover, continuing developments on both the experimental and data-analysis side will require updating of the manuscript before the true added-value can be determined. These requirements, and a number of minor comments, are discussed below. For these reasons, I am currently undecided if the manuscript is a good fit for Genome Biology.

Required updating:

- Although the authors included benchmarking to 5 other tools for TAD hierarchy calling, other tools were not included. An important omission is the "Arrowhead" approach (Rao et al, Cell 2014), which was among the first and still remains one of the most widely used. Moreover, new approaches are regularly presented, which includes for instance the very recent OnTAD approach (An et al, Genome Biology 2019). To allow the most up-to-date comparison, these approaches (and potentially other recent approaches that I may have missed), should be included.

- More problematic is the data set used for benchmarking (Dixon et al, Nature 2012). Technical improvements to the Hi-C protocol, particularly the use of "in-nucleus" ligation (Rao et al, Cell 2014) and highly increased sequencing throughput, provide data sets with much reduced noise levels at the resolutions used for benchmarking (40kb and up). As the very large majority of Hi-C data are generated using this new approach, the added value of SuperTAD becomes obscured. To get a true impression of the value of SuperTAD, the tool should be benchmarked using recent ultra-deep sequenced Hi-C data (similar to the recent introduction of OnTAD):

>> Various human cells lines (including IMR90): Rao et al, Cell 2014

>> Mouse embryonic stem cells + in-vitro differentiated counterparts: Bonev et al, Cell 2017

>> Human embryonic stem cells and fibroblasts (included as control for micro-C data): https://www.biorxiv.org/content/10.1101/639922v1

Considering that these data better represent the current state-of-the-art, they are more informative for benchmarking than the currently used data from Dixon et al.

Minor comments:

1. The main text will profit from some additional detail on the node filtering. Particularly for the non-expert reader, it will be helpful to read in a few words how the optimal TAD size is chosen. In a similar vein, the SuperTAD and deDoc Hi-C matrixes in Figures 5 and 7 will benefit from an indication which level of TADs within the nested structures are selected after filtering (e.g. by using stronger or differently colored lines).

2. Page 1, line 39: The authors mention that many 3C-based techniques have been devised. With the exception of reference 3, all other references are to Hi-C or very similar approaches though.

3. The authors make some generalized comments, which should be nuanced:

- Page 1, line 57: "The size of a TAD varies from thousands of base pairs (kbps) to several million base pairs (the average size is about 900 kbps)". This estimate of median (not average) TAD size is an estimate from one cell type in one paper. Other papers, including the authors here, can be quite different. We should be careful not to turn this number into a gold standard.

- Page 4, line 5: "… deDoc boxes reaches 0 when the noise ratio is equal to 40%". The value starts to approach 0, but even at 50% noise still doesn't reach this number.

4. The numbering of figures is not consistent: figure 1 is references after all other figures. Similarly, only supplemental figure 4 is referenced in the manuscript, whereas the others are mentioned only in the supplemental text.

5. The number of overlapping lines in Figure 6 makes it difficult to access. Could the authors find an alternative or additional measure to better convey the information?

6. Figure 7: the annotation in the matrices is missing.

**Reviewer 2**

**Were you able to assess all statistics in the manuscript, including the appropriateness of statistical tests used?**

No

**Were you able to directly test the methods?**

No

**Comments to author:**

In this manuscript, Zhang et al. describe a new method, called SuperTAD, to identify TAD hierarchies from Hi-C contact matrices. While numerous algorithms for the identification of TADs exist, many suffer from poor reproducibility across different replicate Hi-C maps or at different resolutions, especially for the more finer 'sub-TAD' structures apparent in Hi-C data. SuperTAD is a graph based method that uses structures called coding trees to model the TAD hierarchies. The authors developed and implemented new computationally efficient algorithms to find optimal solutions in terms of structural entropy and applied these to real Hi-C data from 2 human cell lines. They compared their method to five existing methods and show SuperTAD is superior in terms of an accuracy metric called overlapping ratio which they propose and the weighted similarity proposed by Li et al.

Whereas the method proposed is elegant and of potential interest to a broad audience and to study the function of TADs with Hi-C data, it is hard in the current manuscript to properly evaluate how SuperTAD performs in practical applications as compared to existing methods.

Specific comments :

Genome biology published a more comprehensive comparison of 22 computational methods for the identification of topologically associating domains (Zufferey et al. 2018 19:217). Since different assessment criteria and analyses are used by Zhang et al. here, and comparison with some of the top performing methods (such as TopDom, HiCseg) in that paper is lacking, it is hard to compare the performance of SuperTAD to these existing methods. I would recommend to either implement one of the concordance methods used in that paper and evaluate the 5 methods compared here using that measure on the same dataset as was used in Zufferey et al. 2018, or include TopDom and/or HiCseg in addition to the 5 methods tested here.

In Figure 4D, the enrichment of CTCF at the boundaries of the TADs seems much lower than expected, for the hESC data in particular. It would help to put this data into context, by comparing it for instance with the original definition of TADs by Dixon et al. (Nature 2012, doi: 10.1038/nature11082) which reported a much higher enrichment. The same thing is visible in Figure 6 where there is not a very clear enrichment at the boundaries of TADs for most of the methods. Are differences due to a different quantification of the enrichment, or because enrichment in these Hi-C datasets is significantly less or is it because of the definition of the TADs ?

In Table 3 some values are highlighted in red, but not explained why. For TADtree there is no informative data in this Table.

The method currently provides as output lines with numeric indices of consecutive bins belonging to a TAD. For the output to be more generally useful, genomic start and end coordinates of the TADs should be provided.

An example dataset together with a small tutorial / instructions on how to run the SuperTAD method on that dataset should be added.

**Authors Response**

**Point-by-point responses to the reviewers’ comments:**

Reviewer 1:

- Although the authors included benchmarking to 5 other tools for TAD hierarchy calling, other tools were not included. An important omission is the "Arrowhead" approach (Rao et al, Cell 2014), which was among the first and still remains one of the most widely used. Moreover, new approaches are regularly presented, which includes for instance the very recent OnTAD approach (An et al, Genome Biology 2019). To allow the most up-to-date comparison, these approaches (and potentially other recent approaches that I may have missed), should be included.

*We thank the reviewer for this useful comment and agree that adding the Arrowhead and OnTAD algorithm into the comparison would give greater confidence in the overall conclusion that SuperTAD is superior in detecting TAD hierarchies. The Arrowhead algorithm transforms the square domain feature into an arrowhead-shaped feature and determines each domain candidate independently. Therefore its result allows overlap between TADs and is not strictly nested. To adopt Arrowhead into the analysis through structure information theory, we have made some modifications when constructing the Arrowhead tree that we discard the small TADs that are incompatible with the coding tree. Besides, we use all the identified TADs from Arrowhead for the other evaluation. We have addressed this in the manuscript (Page 6, line 4). OnTAD is published the day before our manuscript submission and we have also included OnTAD in the comparison in this revision.*

- More problematic is the data set used for benchmarking (Dixon et al, Nature 2012). Technical improvements to the Hi-C protocol, particularly the use of "in-nucleus" ligation (Rao et al, Cell 2014) and highly increased sequencing throughput, provide data sets with much reduced noise levels at the resolutions used for benchmarking (40kb and up). As the very large majority of Hi-C data are generated using this new approach, the added value of SuperTAD becomes obscured. To get a true impression of the value of SuperTAD, the tool should be benchmarked using recent ultra-deep sequenced Hi-C data (similar to the recent introduction of OnTAD):

>> Various human cells lines (including IMR90): Rao et al, Cell 2014

>> Mouse embryonic stem cells + in-vitro differentiated counterparts: Bonev et al, Cell 2017

>> Human embryonic stem cells and fibroblasts (included as control for micro-C data): https://www.biorxiv.org/content/10.1101/639922v1

Considering that these data better represent the current state-of-the-art, they are more informative for benchmarking than the currently used data from Dixon et al.

*We thank the reviewer for this detailed consideration of the Hi-C benchmarking data. In this revision, we have applied two in situ Hi-C datasets from Rao et al. (Cell 2014). One is the GM12878 dataset, which has 4.9 B contacts, the other is the IMR90 dataset, which has 1.1 B contacts. We downloaded the processed data (in .hic format) and obtained the raw or KR (Knight-Ruiz) normalized contact matrices through Juicer. We used 25 kb for the main analysis and selected 25kb, 50kb, and 100kb to assess the agreement across various resolutions. Our algorithm is proved to be superior in robustness, self-consistency, and biological property testing among the comparison with the other seven methods using the new datasets.*

1. The main text will profit from some additional detail on the node filtering. Particularly for the non-expert reader, it will be helpful to read in a few words how the optimal TAD size is chosen. In a similar vein, the SuperTAD and deDoc Hi-C matrixes in Figures 5 and 7 will benefit from an indication which level of TADs within the nested structures are selected after filtering (e.g. by using stronger or differently colored lines).

*We thank the reviewer for this thoughtful suggestion. We have revised the nodes filtering description in the manuscript to be more clarified and redrew the heatmaps in Supplementary Figure S5 to highlight the selected TADs (colored in red) among all the candidates. As shown in Figure S5 TAD candidates, the most pruned TADs are from level 1 or 2, i.e., the TADs embed no further nesting (level 1) or embed at least one TAD at level 1 (level 2). The filter is provided as an option to be applied to the optimal coding tree (the result of SuperTAD). We have demonstrated that the result after node filtering (SuperTAD(F)) achieves a higher epigenetic characteristic enrichment than SuperTAD while keeping the agreement across various resolutions and cell lines.*

2. Page 1, line 39: The authors mention that many 3C-based techniques have been devised. With the exception of reference 3, all other references are to Hi-C or very similar approaches though.

*We thank the reviewer for pointing this out. We have added three more references in the manuscript. (de Laat W et al. [1] introduced the 3C technology and the subsequent variants 4C, 5C, Hi-C, ChIA-PET, and demonstrated their importance for the understanding of the genome structure and function. Han et al. [2] described and compared the methodologies of 3C and 3C-derived techniques. Übelmesser et al. [3] introduced the most recent advances in technology for eliminating systematic biases and increase precision.)*

3. The authors make some generalized comments, which should be nuanced:

- Page 1, line 57: "The size of a TAD varies from thousands of base pairs (kbps) to several million base pairs (the average size is about 900 kbps)". This estimate of median (not average) TAD size is an estimate from one cell type in one paper. Other papers, including the authors here, can be quite different. We should be careful not to turn this number into a gold standard.

- Page 4, line 5: "… deDoc boxes reaches 0 when the noise ratio is equal to 40%". The value starts to approach 0, but even at 50% noise still doesn't reach this number.

*We appreciate the reviewer’s comments. In the revised manuscript, we have discarded the statement of referenced average TAD sizes.*

*The green points in boxes indicate the mean value, while the solid black line indicates the median value. When the noise ratio equals to 40%, there is no solid line in the deDoc’s boxes as the median value turns to zero while the mean value decreases to a small value but still larger than zero. We have corrected the annotation of the median value to clarify this trend.*

4. The numbering of figures is not consistent: figure 1 is references after all other figures. Similarly, only supplemental figure 4 is referenced in the manuscript, whereas the others are mentioned only in the supplemental text.

*We thank the reviewer for this suggestion. We have corrected the order of figures in the manuscript. We have added references for all the tables and figures in supplementary in this revision (Table S1: Page 8, the end of paragraph 4; Figure S1: Page 7, paragraph 3; Figure S2: Page 11, highlight; Figure S3: Page 12, highlight; Figure S4, S5: page 13, the end of paragraph 1).*

5. The number of overlapping lines in Figure 6 makes it difficult to access. Could the authors find an alternative or additional measure to better convey the information?

*We thank the reviewer for this useful comment and have revised the analysis to quantify the enrichment of epigenetic characteristics. The enrichment analyses of epigenetic characteristics are in two parts.*

*As for structural proteins CTCF, RAD21, and SMC3, we compute the average peaks around the TAD boundaries (+-1 bin, referred to as peak) and the region located 400 kb away from the boundaries with the length of 100 kb at both sides (referred as background). Then we compute the fold change between peak and background to quantify the enrichment. A positive fold change indicates the boundaries have an enrichment of the proteins.*

*As for repressing/activating histone modifications H3K36me3 and H3K27me3, we evaluate the enrichment of ChIP-seq signals inside the TADs. A TAD can be assigned to enriched for either H3K36me3 or H3K27me3, or neither under FDR correction. We calculate the fractions of TADs enriched for each of the histone modifications and compare them among methods. The higher fraction of TADs enriched for histone marks demonstrates the TADs predicted by the algorithm exhibiting more biological properties.*

6. Figure 7: the annotation in the matrices is missing.

*We have added the annotation for the boundaries drawn on the upper triangle and the lower triangle of the heatmaps in the revision.*

Reviewer 2:

Genome biology published a more comprehensive comparison of 22 computational methods for the identification of topologically associating domains (Zufferey et al. 2018 19:217). Since different assessment criteria and analyses are used by Zhang et al. here, and comparison with some of the top performing methods (such as TopDom, HiCseg) in that paper is lacking, it is hard to compare the performance of SuperTAD to these existing methods. I would recommend to either implement one of the concordance methods used in that paper and evaluate the 5 methods compared here using that measure on the same dataset as was used in Zufferey et al. 2018, or include TopDom and/or HiCseg in addition to the 5 methods tested here.

*We thank the reviewer for this thoughtful comment. We have determined to accept the reviewer's first suggestion as our manuscript focused on detecting the TAD hierarchy while both TopDom and HiCseg are the good tools for detecting disjoint TADs. In this revision, we compared SuperTAD with seven state-of-the-art algorithms (Arrowhead and OnTAD are added in the comparison). All of the selected algorithms are proposed to identify the TADs in a nested manner except Arrowhead, which results is not strictly nested. We have implemented the MoC in our manuscript to assess the algorithms' agreement, along with overlapping ratio and weighted similarity. We have applied the same dataset as Zufferey et al., in situ Hi-C data for GM12878 cell lines with an ultra-deep sequence-depth along with other human cell lines, IMR90 cells.*

In Figure 4D, the enrichment of CTCF at the boundaries of the TADs seems much lower than expected, for the hESC data in particular. It would help to put this data into context, by comparing it for instance with the original definition of TADs by Dixon et al. (Nature 2012, doi: 10.1038/nature11082) which reported a much higher enrichment. The same thing is visible in Figure 6 where there is not a very clear enrichment at the boundaries of TADs for most of the methods. Are differences due to a different quantification of the enrichment, or because enrichment in these Hi-C datasets is significantly less or is it because of the definition of the TADs ?

*The differences might be due to two reasons. One is the insufficient read depth of ChIP-seq data, and the other is the original enrichment analysis lack of quantification to depict the results better. In this revision, we collected all the available ChIP-seq data for GM12878 and IMR90 datasets from ENCODE(https://www.encodeproject.org/). We took the intersection to merge the optimal IDR thresholded peaks across various experiments and applied the merged peaks for fold change analysis.*

*To address the second issue, we referenced the analysis from Zufferey et al. 2018 19:217 and quantified the enrichment of epigenetic characteristics around TAD boundaries or within TADs through computing the fold change or the FDR corrected p-value. We replaced Figure 4D and Figure 6 with a bar chart and accumulative bar diagram (Figure 3E, F, and Figure 5 in this revision) to better convey the result. The TAD boundaries are defined as the ends of TADs as [4]. Our result shows a similar enrichment as Zufferey et al.*

In Table 3 some values are highlighted in red, but not explained why. For TADtree there is no informative data in this Table.

*We thank the reviewer for this thoughtful reminder. We have added the footnotes for both issues in the revision. We labeled the top two largest values in red for each line in Table 3 to highlight the excellent performance of the self-consistency of methods. We also explained that the nan value is due to the comparison lack one or two results for the method in the footnote of Tables 2 and 3.*

The method currently provides as output lines with numeric indices of consecutive bins belonging to a TAD. For the output to be more generally useful, genomic start and end coordinates of the TADs should be provided.

*We thank the reviewer for this thoughtful comment and have added the genomic coordinate information in output. We have also adopted the uniform eight-column format when analyzing and comparing all the methods. The format records the bin index and genomic coordinate of identified boundaries; each column is separated by a tab. Take one TAD for example (at 25 kb bin resolution):*

*chr1 1 0 25000 chr1 20 475000 500000*

*Each column is represented as:*

*1st the chromosome of left boundary*

*2nd the bin index that identified as the left boundary (start bin)*

*3rd the start coordinate of start bin, in bp*

*4th the end coordinate of start bin, in bp*

*5th the chromosome of right boundary*

*6th the bin index that identified as the right boundary (end bin)*

*7th the start coordinate of end bin, in bp*

*8th the end coordinate of end bin, in bp*

An example dataset together with a small tutorial / instructions on how to run the SuperTAD method on that dataset should be added.

*We thank the reviewer for this kind reminder. We have revised the README file and added the examples on how to run SuperTAD with example datasets. Also you may find detailed instructions on the compilation, usage, data format, etc. in the README. SuperTAD is available at our GitLab repository(https://gitlab.deepomics.org/mengbo/SuperTAD) or https://supertad.deepomics.org/. The version used in the manuscript are permanently available at https://doi.org/10.5281/zenodo.3968226.*

*References*

*[1] de Laat W, Dekker J. 3C-based technologies to study the shape of the genome[J]. Methods (San Diego, Calif.), 2012, 58(3).*

*[2] Han J, Zhang Z, Wang K. 3C and 3C-based techniques: the powerful tools for spatial genome organization deciphering[J]. Molecular Cytogenetics, 2018, 11(1): 1-10.*

*[3] Übelmesser N, Papantonis A. Technologies to study spatial genome organization: beyond 3C[J]. Briefings in Functional Genomics, 2019, 18(6): 395-401.*

*[4] Hong S, Kim D. Computational characterization of chromatin domain boundary-associated genomic elements[J]. Nucleic acids research, 2017, 45(18): 10403-10414.*

**Second round of review**

**Reviewer 1**

In this revised version of the manuscript, the authors have sufficiently addressed most issues. Particularly the use of higher resolution/higher sequencing depth Hi-C data greatly helps to better assess the quality of SuperTAD relative to other tools.

The revised manuscript includes a new analysis that raises several questions though:

On page 5 and 6, and accompanying figures 3F and 5D, the enrichment of histone modifications within TADs is determined.

-	First of all, the rationale for this analysis is not well described in the text.

-	Assuming that the authors want to show that SuperTAD is better able to separate euchromatic TADs from heterochromatic TADs, one may ask if the H3K27me3 histone mark is most appropriate. Previous studies have suggested that the H3K27me3 mark occupies an intermediate position, in-between true euchromatin and heterochromatin (Rao et al, Cell 2014 – Fig. 2D and F (as compared to the somewhat misleading Fig. 2A from the same publication); Vieux-Rochas et al, PNAS 2015 – Fig. 2B; Bonev et al, Cell 2017 – Fig. 1G). A more appropriate analysis would incorporate the active H3K36me3 mark, optionally the H3K27me3 mark, and the truly repressive H3K9me3 mark. This latter mark is surely available for the GM12878 cell type (see Rao et al, Cell 2014). As an alternative, the authors could also correlate TAD boundaries to the boundaries of ‘Hi-C compartments A and B’, which globally separate euchromatin (compartment A) and heterochromatin (compartment B; see Lieberman-Aiden, Science 2009 and Dixon et al, Nature 2012).

-	The green bar in the legend for figures 3F and 5D mentions FDR > 0.1, whereas the other bars indicate histone modifications. This appears not correct to me.

Additionally, I have two minor comments:

-	Figure 3FG and Figure 6 are interpreted without actual quantitation (or as the authors state “Intuitively, SuperTAD, SuperTAD(F), and deDoc are relatively consistent…” (page 7, line 18). The interpretation of these figures will be improved by adding a factual quantitation of the result.

-	Page 4, line 62: “members of cohesin”. Biochemically it is more correct to mention “members of the Cohesin protein complex”.

**Reviewer 2**

The authors did a great job in the revision and sufficiently addressed my previous comments and suggestions. The manuscript has been significantly improved. I have just a few last minor comments.

Figure 3 :

Legend : There is an error in the description / labeling of the panels in the Figure legend. Where it reads F,G it should read G,H (heatmap panels).

Figure 5 legend : accumulative bar diagram should be cummulative bar diagram

**Authors Response**

**Point-by-point responses to the reviewers’ comments:**

*We thank the reviewers for their generous comments on the manuscript GBIO-D-19-01951R1 entitled “SuperTAD: Robust detection of hierarchical topologically associated domains with the optimized structural information”.*

*We have carefully addressed the newly proposed comments. Also, we included a detailed point-by-point response to all the comments.*

*We believe that the manuscript is now suitable for publication in Genome Biology.*

Review point 1.1

"On page 5 and 6, and accompanying figures 3F and 5D, the enrichment of histone modifications within TADs is determined.

- First of all, the rationale for this analysis is not well described in the text. "

*We thank the reviewer for pointing this out. The rationale behind this analysis is that TADs are frequently enriched for either activating (H3K36me3) or repressing (H3K27me3) marks, which indicate transcriptional activity within domains. Therefore, we want to assess and compare the fraction of TADs significantly enriched for either mark across methods. We have added the rationale of this analysis at the beginning of paragraph 2, Page 5, in the revised manuscript.*

“- Assuming that the authors want to show that SuperTAD is better able to separate euchromatic TADs from heterochromatic TADs, one may ask if the H3K27me3 histone mark is most appropriate. Previous studies have suggested that the H3K27me3 mark occupies an intermediate position, in-between true euchromatin and heterochromatin (Rao et al, Cell 2014 – Fig. 2D and F (as compared to the somewhat misleading Fig. 2A from the same publication); Vieux-Rochas et al, PNAS 2015 – Fig. 2B; Bonev et al, Cell 2017 – Fig. 1G). A more appropriate analysis would incorporate the active H3K36me3 mark, optionally the H3K27me3 mark, and the truly repressive H3K9me3 mark. This latter mark is surely available for the GM12878 cell type (see Rao et al, Cell 2014). As an alternative, the authors could also correlate TAD boundaries to the boundaries of ‘Hi-C compartments A and B’, which globally separate euchromatin (compartment A) and heterochromatin (compartment B; see Lieberman-Aiden, Science 2009 and Dixon et al, Nature 2012). “

*We want to thank the reviewer for this detailed consideration of our histone modification enrichment analysis within TADs and apologize for our unclear descriptions that lead to this misunderstanding. Through this analysis, what we want to show is that the SuperTAD result exhibits a higher percentage of TADs that have significant enrichment of either activating or repressing marks, which reflects a more biological meaningful result of the algorithm.*

*To conduct this analysis, we downloaded the ChIP-seq signals (fold change over control data) for H3K36me3 and H3K27me3 from ENCODE and calculated the observed average log10 ratio (LR) of H3K27me3/H3K36me3 and the empirical $p$-value derived from 1000 times shuffling for each TAD. Based on the FDR corrected $p$-value, we \hl{identified the TADs that significantly enriched for either mark (FDR corrected $p$-value $\le 0.1$) from those with no significant enrichment (FDR corrected $p$-value $> 0.1$). Then we further divide the TADs from the former group (FDR corrected $p$-value $\le 0.1$) into two sets, one is enriched in H3K27me3 and the other is enriched in H3K36me3, based on the observed LR value for each TAD, while this step is not our main purpose.*

*The reasons why we choose the activating mark H3K36me3 and repressing mark H3K27me3 (instead of H3K9me3) to conduct the enrichment analysis are as follows:*

*1. The two histone modifications show a good mutual exclusion. Rao et al. have proved that A1 and A2 contact domains are gene dense and linked to active chromatin marks (enriched in H3K36me3). B1, B2, B3, and B4 sub-compartments are linked to repressive chromatin. B1 contact domains are linked to facultative heterochromatin (correlated positively with H3K27me3 and negatively with H3K36me3), while the B2 and B3 tend to lack all the marks and enriched at the pericentromeric heterochromatin and nuclear lamina. The sub-compartment B4 is restricted to a small 11 Mb portion of chromosome 19 containing both active chromatin (i.e., H3K36me3) and heterochromatin-associated (i.e., H3K9me3) marks [1]. Similar patterns are also found in Drosophila [2]. Therefore H3K27me3 is more appropriate to distinguish the repressed transcriptional domains from active transcriptional domains (mainly in A1, A2, and B1).*

2. TADs are frequently enriched for these two histone marks. The transcriptionally active domains in both human and Drosophila are positively associated with H3K36me3, which particularly comprise 42% of the domains and 22% of the genome in Drosophila [2]. Nora et al. found that TADs exhibited a striking alignment with the large blocks of H3K27me3 [3]. As B2 and B3 are enriched at the pericentromeric heterochromatin and nuclear lamina, H3K36me3 and H3K27me3 can indicate either active or repressed transcriptional domains on the well mappable part of the genome.

“- The green bar in the legend for figures 3F and 5D mentions FDR > 0.1, whereas the other bars indicate histone modifications. This appears not correct to me. “

*We thank the reviewer for this useful comment and apologize again for our unclear descriptions of this. Based on the FDR corrected p-value, we first identified the TADs that exhibited significant enrichment for either mark (FDR <= 0.1, the sum of the blue and orange bar) from those with no significant enrichment (FDR > 0.1, the green bar). Then, based on the observed LR value of H3K27me3/H3K36me3, we further divided the former group (FDR <= 0.1) into two sets, one is enriched in H3K27me3 (the blue bar), the other is enriched in H3K36me3 (the orange bar). The fractions of the TADs with no histone marks enriched (FDR > 0.1) and the two sets are calculated and displayed as the bar height in figures 3F and 5CD. To avoid the misleading, we have clarified this in the revised manuscript (paragraph 2, Page 5; paragraph 1, Page 7; paragraph 2, Page 9; the legend of Figure 3F, Page 18; the legend of Figure 5CD, Page 19).*

Review point 1.2

“Figure 3FG and Figure 6 are interpreted without actual quantitation (or as the authors state “Intuitively, SuperTAD, SuperTAD(F), and deDoc are relatively consistent…” (page 7, line 18). The interpretation of these figures will be improved by adding a factual quantitation of the result.”

*We thank the reviewer for this valuable suggestion. The quoted sentence and the rest of the same paragraph are our descriptions of the heatmaps in Figure 6. In the heatmap, we aimed to provide an intuitive similarity performance between various resolutions across methods. Then we quantified the similarity though overlapping ratio, weighted similarity, and MoC to further demonstrate that our method exhibited consistent and high similarity between various resolutions across cell lines. In the revised manuscript, we have deleted the positive reviews of SuperTAD, SuperTAD(F), and deDoc in Figure 6 as no quantified assessment combined.*

Review point 1.3

“Page 4, line 62: “members of cohesin”. Biochemically it is more correct to mention “members of the Cohesin protein complex”.”

*We thank the reviewer for this considerate correction. We have corrected the statement in the revised manuscript.*

Review point 2.1

"Figure 3 Legend : There is an error in the description / labeling of the panels in the Figure legend. Where it reads F,G it should read G,H (heatmap panels)."

*We thank the reviewer for this thoughtful correction. We have corrected the error in the revised manuscript.*

Review point 2.2

"Figure 5 legend : accumulative bar diagram should be cummulative bar diagram."

*We thank the reviewer for this thoughtful correction. We have changed as suggested.*

*Reference*

*1. Rao S S P, Huntley M H, Durand N C, et al. A 3D map of the human genome at kilobase resolution reveals principles of chromatin looping[J]. Cell, 2014, 159(7): 1665-1680.*

*2. Ea V, Baudement M O, Lesne A, et al. Contribution of topological domains and loop formation to 3D chromatin organization[J]. Genes, 2015, 6(3): 734-750.*

*3. Nora E P, Lajoie B R, Schulz E G, et al. Spatial partitioning of the regulatory landscape of the X-inactivation centre[J]. Nature, 2012, 485(7398): 381-385.*

**Third round of review**

**Reviewer 1**

I thank the reviewers for the detailed replies to my questions and the associated modifications to the manuscript. These further explanations provide important help for the interpretation of the manuscript.

With these further modifications / clarifications, I now consider the manuscript overall suitable for publication in Genome Research. I just would like to propose two minor textual edits, to avoid confusion:

1. The term 'histone-3' is used several times throughout the manuscript. The common writing in the epigenetics and chromatin field is 'Histone H3' (including capitalization: see https://en.wikipedia.org/wiki/Histone_H3). The authors should change this.

2. I'm coming back to the term 'Intuitively' on page 7. My initial comment was to point out that this word has negative connotation: it indicates that the interpretation is not based on scientific reason. Based on the explanation by the authors, I propose to replace the word by 'As can be observed,...'
